# Transcriptome-wide association mapping provides insights into the genetic basis and candidate genes governing flowering, maturity and seed weight in rice bean (*Vigna umbellata*)

**DOI:** 10.1186/s12870-024-04976-y

**Published:** 2024-05-08

**Authors:** Tanmaya Kumar Sahu, Sachin Kumar Verma, Gayacharan, Nagendra Pratap Singh, Dinesh Chandra Joshi, D. P. Wankhede, Mohar Singh, Rakesh Bhardwaj, Badal Singh, Swarup Kumar Parida, Debasis Chattopadhyay, Gyanendra Pratap Singh, Amit Kumar Singh

**Affiliations:** 1 ICAR-National Bureau of Plant Genetic Resources, Pusa Campus, New Delhi, 110012 India; 2 ICAR-Indian Grassland and Fodder Research Institute, Jhansi, Uttar Pradesh India; 3 National Institute of Plant Genome Research, Aruna Asaf Ali Marg, New Delhi, India; 4 ICAR-Vivekananda Parvatiya Krishi Anusandhan Sansthan, Almora, Uttarakhand India

**Keywords:** GWAS, Rice bean, WRKY1, DEAD box RH27, Phospholipid transporting ATPase-9, Aldo–keto reductase, HSC80, P-II PsbX

## Abstract

**Background:**

Rice bean (*Vigna umbellata*), an underrated legume, adapts to diverse climatic conditions with the potential to support food and nutritional security worldwide. It is used as a vegetable, minor food crop and a fodder crop, being a rich source of proteins, minerals, and essential fatty acids. However, little effort has been made to decipher the genetic and molecular basis of various useful traits in this crop. Therefore, we considered three economically important traits *i.e.,* flowering, maturity and seed weight of rice bean and identified the associated candidate genes employing an associative transcriptomics approach on 100 diverse genotypes out of 1800 evaluated rice bean accessions from the Indian National Genebank.

**Results:**

The transcriptomics-based genotyping of one-hundred diverse rice bean cultivars followed by pre-processing of genotypic data resulted in 49,271 filtered markers. The STRUCTURE, PCA and Neighbor-Joining clustering of 100 genotypes revealed three putative sub-populations. The marker-trait association analysis involving various genome-wide association study (GWAS) models revealed significant association of 82 markers on 48 transcripts for flowering, 26 markers on 22 transcripts for maturity and 22 markers on 21 transcripts for seed weight. The transcript annotation provided information on the putative candidate genes for the considered traits. The candidate genes identified for flowering include HSC80, P-II PsbX, phospholipid-transporting-ATPase-9, pectin-acetylesterase-8 and E3-ubiquitin-protein-ligase-RHG1A. Further, the WRKY1 and DEAD-box-RH27 were found to be associated with seed weight. Furthermore, the associations of PIF3 and pentatricopeptide-repeat-containing-gene with maturity and seed weight, and aldo–keto-reductase with flowering and maturity were revealed.

**Conclusion:**

This study offers insights into the genetic basis of key agronomic traits in rice bean, including flowering, maturity, and seed weight. The identified markers and associated candidate genes provide valuable resources for future exploration and targeted breeding, aiming to enhance the agronomic performance of rice bean cultivars. Notably, this research represents the first transcriptome-wide association study in pulse crop, uncovering the candidate genes for agronomically useful traits.

**Supplementary Information:**

The online version contains supplementary material available at 10.1186/s12870-024-04976-y.

## Introduction

The rice bean [*Vigna umbellata* (Thunb.) Ohwi & Ohashi] is a relatively short-duration and an annual warm-season leguminous pulse crop. It is reported to be able to adapt to a diverse range of climatic conditions and can be grown in a wide range of soil types, even in the poor quality soil [1]. It is used as a vegetable, a minor food crop, a fodder crop and a green manure as well. Being a leguminous crop, rice bean has an advantageous role in mixed cropping and preventing soil erosion due to its ability to fix nitrogen in nutrient depleted soils [2]. Rice bean is reportedly resistant to a wide range of biotic and abiotic stresses. Especially it is a source of resistance against biotic stresses such as bruchids, yellow mosaic disease and bacterial leaf spots [3]. Among abiotic stresses, it is tolerant to some degree of water logging [4], acid soils [5], drought [6] and high temperatures.

Rice bean is important for the health of the dependent living beings as well as the soil. It has great potential to overcome food and nutritional deficiency across the globe [7]. In favorable climatic conditions, it produces large amounts of healthy animal fodder and superior quality grains. It is a rich source of proteins, minerals, essential fatty acids and amino acids [8]. Its amino acid composition is reportedly well balanced for human consumption [4, 8, 9]. Though it is a beneficial, nutritious and low maintenance crop, little research has been carried out on it. It has also remained as a neglected crop being cultivated on small areas in India, Nepal and parts of Southeast Asia [2, 10, 11]. Due to this negligence, it suffers from wild and disadvantageous traits such as indeterminate growth habit, asynchronous and late maturity, pod shattering and anti-nutritional compounds [7]. Additionally, the level of enzyme inhibitors, anti-nutritive or toxic factors are low in rice bean compared to other legumes [12].

Rice bean is a diploid crop with 11 pairs of chromosomes (2n = 22) and has an estimated genome size of ~ 525.60 Mb [13]. The availability of inadequate genomic information on rice bean has made the investigations on this crop challenging. A few genetic maps of rice bean have been constructed and utilized to localize genes for quantitative traits such as seed weight and several domestication related traits [3, 14]. However, these maps do not provide high resolution mapping of targeted traits because they were based on a small number of simple sequence repeat (SSR) markers derived from the related legume species [3]. Very recently in September 2022, one genome assembly of rice bean at the chromosomal level has been released by Chinese Academy of Agricultural Sciences, Beijing, China [13] with the genome size of 475.64 Mb that is 90.49% of the estimated genome size. However, another assembly by International Centre for Genetic Engineering and Biotechnology, New Delhi, India is available at scaffold level [15] having the assembled genome size of 414 Mb. They estimated a total of 31276 highly confidential genes from 15521 scaffolds. Availability of these two assemblies opened an avenue for annotating markers and the corresponding candidate genes for important traits revealed through genome or transcriptome wide association analysis.

The cultivars of rice bean are highly photoperiod sensitive. Therefore, when the crop is grown in subtropical areas, vegetative growth continues for a longer duration and the crop flowers very late. Further, the rice bean crop improvement and its cultivation faces a challenge from other *Vigna* group of crops of similar nature but with superior agronomic traits. The yield potential of rice bean is estimated to be 1200 kg/ha [16], which is comparatively lesser than green gram, black gram and cowpea. Similarly, indeterminate growth habit and pod shattering make the crop unsuitable for large scale or mechanized farming primarily grown as intercrop [16]. Owing to such constraints and the competition from other crops, rice bean cultivation area has gradually decreased and is even discontinued from traditional cultivation areas. However, with the recent trend towards food crop diversification amid climate change and health awareness of the society, rice bean crop is recognized as one of the most potential legume crops to meet out the current and future needs. Therefore, we selected the three most important productivity related traits i.e., days to flowering, days to maturity after crop sowing and seed size, to understand their genetics. Understanding the underlying genetic mechanism governing these traits will help in developing rice bean genotypes with early flowering, early maturity, and bold seeds, which will enhance the large-scale area expansion under rice bean crop.

Genome-wide association studies (GWAS) involving genotypic and phenotypic data has empowered efficient detection of significant markers associated with the trait of interest [17, 18]. If interest lies in the expressed part of the genome, variant calling from transcriptome sequencing can be adopted, which is comparatively easier, cost effective and time efficient than whole genome sequencing [19, 20]. Further, transcriptome-based variant calling is expected to capture the variants in the parts of the transcripts modified during post-transcriptional modifications and RNA editing. Therefore, we attempted here transcriptome-based variant calling along with association analysis to unravel the putative candidate genes for important productivity related traits like flowering, maturity and seed weight of rice bean. The comprehensive associative transcriptome analysis on rice bean explored here is expected to enlighten the stakeholders on the implementation of transcriptome-based variant calling to identify putative candidate genes for economically important traits in other crops, too.

## Materials and methods

### Plant material

**Fig. 1:**
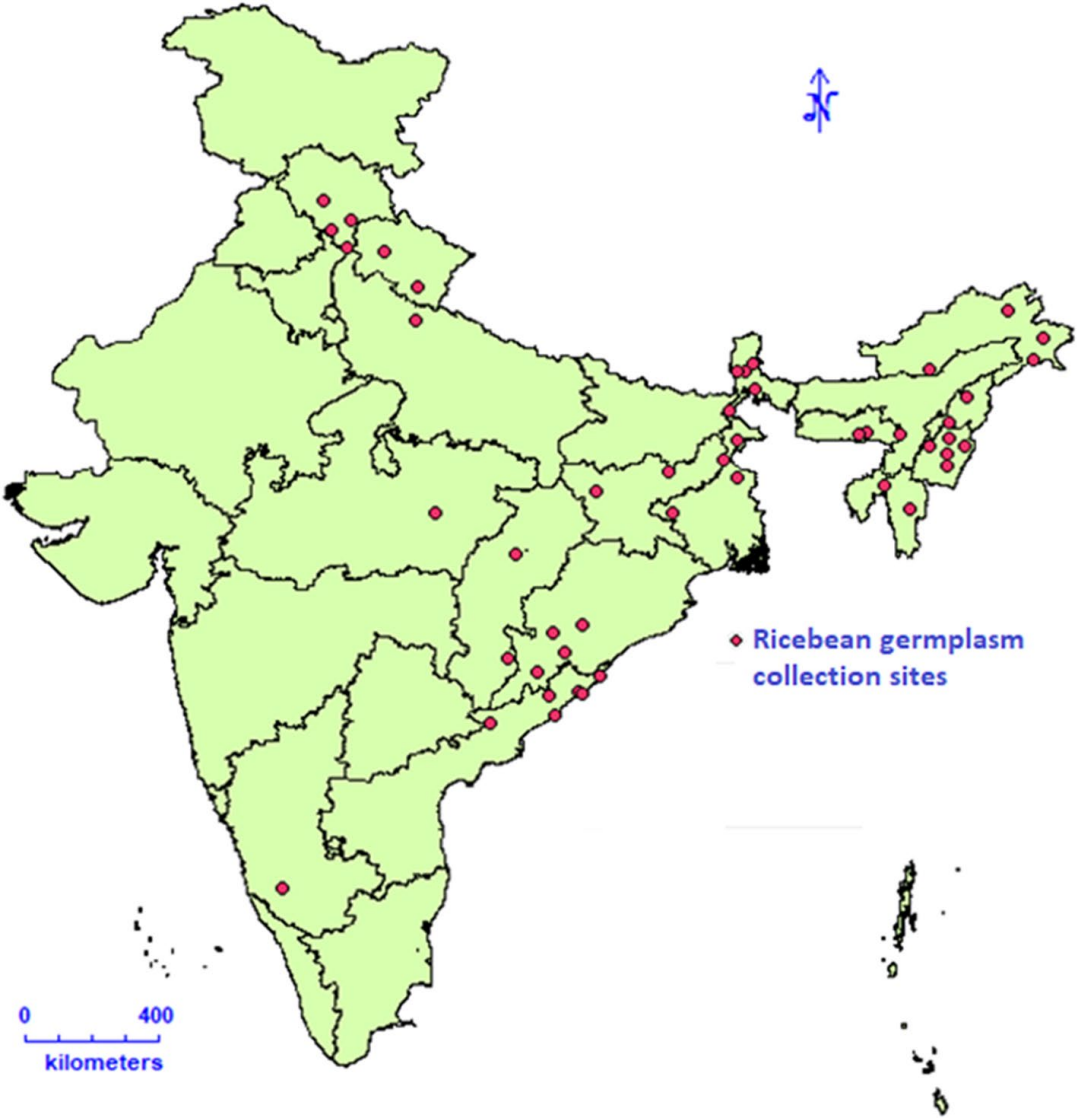
Geographical distribution of 100 rice bean accessions. The map used in this figure has been created using the diva-gis (https://www.diva-gis.org/) web based software. The red points on the map are the site of collection for the genotypes under study

A diverse set of 100 accessions of rice bean was selected based on the phenotypic characteristics of 1800 accessions, which were acquired from National Genebank of India (http://pgrportal.nbpgr.ernet.in/) and characterized for important agro-morphological traits during 2018 and 2019. We also tried to make a selection of these accessions in such a way that each rice bean growing geographical region of India is represented (Fig. 1). To avoid any admixture, a single plant selection representative to the accession population was made. Based on the phenotypic characteristic information generated from the entire set of rice bean accessions, 100 accessions were selected, having a good range of variation for flowering and maturity period and seed size. The selected 100 accessions were further grown during the rainy season of years 2020 and 2021 at the Experimental Farm of ICAR-National Bureau of Plant Genetic Resources, Issapur (28° 34′25″N, 76°50′41″E, 215 m msl) and Experimental Farm Hawalbagh, ICAR-Vivekananda Institute of Hill Agriculture, Almora (79.39° E longitude and 25.35° N latitude, mean rainfall-1000 mm and 1250 m above msl) following augmented block design. Each accession was sown in paired rows of 2 m in length and a row-to-row distance of 60 cm, and a plant to plant distance of 30 cm. The standard rice bean growing practices were followed to obtain the ideal phenotypic expression of genotypes. The detailed passport information about these accessions, such as date of collection, site of origin and cultivar name, is given in Table S1. The phenotypic data for 100 accessions, including days to 50% flowering, days to 80% maturity and 100-seed weight were recorded for all the accessions.

### Data acquisition and processing

#### Phenotypic data analysis

The phenotypic data collected from two locations in two consecutive years was analyzed, and significant variations in the datasets were detected through analysis of variance (ANOVA) for each trait in the interface of SPSS version 15. The frequency distributions and trait-wise comparative density plots were generated using the *sm* package [21] of R.

#### Genotypic data generation

The transcriptome sequencing of the considered genotypes was carried out in the Illumina Hiseq6000 platform. Single-nucleotide polymorphisms (SNPs) were identified using GATK version v4.1 by mapping cleaned short reads (obtained by fastp v0.12) against the rice bean transcriptome reference VRB3 [22] using BWA-mem v0.7 [23]. Duplicate reads were marked by Picard tools (https://broadinstitute.github.io/picard/) and genomic variants were identified using GATK HaplotypeCaller [24]. A joint variant call set was generated using GATK Genotype GVCFs, and subsequently, the SNP variants were selected and filtered to retain high-quality SNPs.

#### Pre-processing of genotypic data

The genotypic data was pre-processed in the interface of TASSEL software (Trait Analysis by Association, Evolution and Linkage version 5. 2. 85) [25]. Initially, the markers were filtered out based on minor allele frequency (MAF > 5%), missing data (< 20%) and heterozygosity (< 50%). As rice bean is a cross-pollinated crop the heterozygosity filter of 50% was implemented. Further, indels, triallelic markers and markers with minor states were removed. The genotypes were also examined for > 30% missing data and > 50% heterozygosity.

### Population structure and diversity

To estimate the number of populations, the genotypic data was subjected to principal component analysis (PCA) and population structure analysis. The PCA was carried out in the graphical user interface of TASSEL on the filtered set of markers, and the first three principal components were plotted to analyze the population structure. To execute the admixture model of STRUCTURE v2.3.4 [26] with Bayesian Markov Chain Monte Carlo model (MCMC) simulation, the markers having high polymorphic information content (PIC) and genetic diversity (GD) were considered. The PIC and GD for each marker were computed based on the following formulae:

$$GD = 1 - \sum\limits_{i = 1}^{2} {a_{i}^{2} }$$

and,

$$PIC = GD - 2a_{1}^{2} a_{2}^{2}$$

where, *a*_*i*_ (*i* = 1, 2) is the frequency of *i*^th^ allele in the population. Here, the value of *i* varies from 1 to 2, because we have considered only the bi-allelic markers. The markers with PIC value >= 0.35 were selected for STRUCTURE analysis with the parameters: K = 2–7, 25000 burnin, 50000 MCMC iterations and ten independent runs. The best value of K was determined using Structure Harvester [27] and the corresponding population membership file was used as the Q-matrix for the association analysis.

### Linkage disequilibrium analysis

The linkage disequilibrium (LD) decay analysis was carried out in the interface of TASSEL to examine the genomic distance within which the genomic elements are believed to have strong LD. As we have used transcriptome data, short-distance LD has been examined by plotting the frequency of the squared allele of LD (r^2^) (Y-axis) against distance in base pair (X-axis) in the R command line for all pair-wise comparisons.

### Association analysis

The association analysis of markers with the considered traits was carried out using two single locus models (with two variations each) and six multi-locus models. Among the single locus models, generalized linear models (GLM) [28] and mixed linear models (MLM) [29, 30] have been used. Two variants each of GLM [GLM (Q) and GLM (PCA)] and MLM [MLM (Q + K) and MLM (PCA + K)] have been used where Q involves the population membership information (Q matrix) as covariates and PCA involves first three principal components (PC) as covariates in the models. The K in MLM refers to the kinship matrix (K) generated based on identity-by-state analysis. These models were implemented using TASSEL, GAPIT [31] and mrMLM.GUI package [32].

Six multi-locus models viz., multi-locus random-SNP-effect MLM (mrMLM) [33], fast multi-locus random-SNP-effect MLM (FASTmrMLM) [34], fast multi-locus random-SNP-effect EMMA(FASTmrEMMA) [32], Iterative Sure Independence Screening EM-Bayesian LASSO (ISIS EMBLASSO) [35], fixed and random model circulating probability unification (FarmCPU) [36] and Bayesian-information and linkage-disequilibrium iteratively nested keyway (BLINK) [37] were used in the study to analyze the marker-trait associations. The FarmCPU and BLINK were implemented through GAPIT package whereas all other multi-locus models were executed through mrMLM.GUI package in R.

### Marker selection

Markers have been selected based on different parameters for thresholds in different software packages. For the single locus models implemented through TASSEL, the markers were selected based on “–log10 (p)” value > 5.99 after Bonferroni correction [38] *i.e.,* 0.05/total number of markers. In the case of multi-locus models implemented through GAPIT, the markers were also selected based on “–log10(p)” value > 3.69(*i.e.,* 2e-4) [39]. However, in mrMLM a separate marker screening parameter viz*,* logarithm of the odds (LOD) score was used. Here, markers were considered to be associated with a trait of interest if they had the LOD score > 3 [39]. Though the initial screening of the markers was based on the above defined parameters, the final screening was on the basis of their association predicted by at least two GWAS models.

### Candidate gene identification

The transcripts on which the significant markers are located were subjected to a BLAST (Basic Local Alignment Search Tool) search for identifying the corresponding genes. To perform the BLAST search, the transcript sequences were aligned to a local protein sequence database using the blastx program of the offline BLAST. For creating a local database, the protein sequences of *Vigna*, G*lycine max* and *Arabidopsis* genus were collected from the National Center for Biotechnology Information (NCBI), USA. As the latter two plant species are well annotated, and *G. max* is the close relative of rice bean apart from other *Vigna* members, the protein sequences of these two plants were included to develop the local BLAST database. Further, all the transcripts were subjected to BLAST2GO of OmicsBox tool (https://www.biobam.com/omicsbox/) for annotation with gene ontology (GO) terms.

### Chromosomal localization of associated markers and transcript synteny

To unravel the putative chromosomal location of markers as well as candidate genes, 50 bp left and 50 bp right flanking to the markers were extracted using a developed R- script and the resulting 101 bp fragments were subjected to the offline blastn program against the recently released fully sequenced genome of *V. umbellata* cultivar FF25 [13]. The exact marker locations were identified from the ungapped alignment of SNP sequences and chromosomal sequences obtained through blastn program. A chromosomal map of associated markers was created based on the newly released fully sequenced genome of *V. umbellata* in the interface of MapChart [40]. Further, full length transcripts were subjected to blastn locally, against the genomes of *V. radiata, V. angularis, V. mungo, V. unguiculata* and *V. umbellata* to identify their corresponding chromosomal locations. The resulting chromosomal coordinates were used to carry out a synteny analysis using ShinyCircos software [41].

### Expression analysis of associated transcripts

The expression of genes related to flowering, maturity and seed weight represented by the associated transcripts was checked in transcriptome data of inflorescence and developing seed tissues. The RNA from the two different developing seed stages i.e., 5-days post anthesis (SRR16122607) and 10-days post anthesis (SRR16122602) of rice bean (accession: IC426787) was sequenced on Illumina HIseq4000. Additionally, the RNA sequencing reads of rice bean samples at the young inflorescence stage (SRR5764826) were also downloaded from NCBI. These reads were processed using FastQC (version 0.11.9) [42], Trimomatic(version 0.40) [43], bwa [44] and samtools [45] for quality check, trimming, mapping and obtaining FPKM (fragments per kilobase of exon per million mapped fragments) values, respectively. A heatmap of the expression pattern was generated using an R-script with heatmap() function.

## Results

### Variation in phenotypic data

**Fig. 2:**
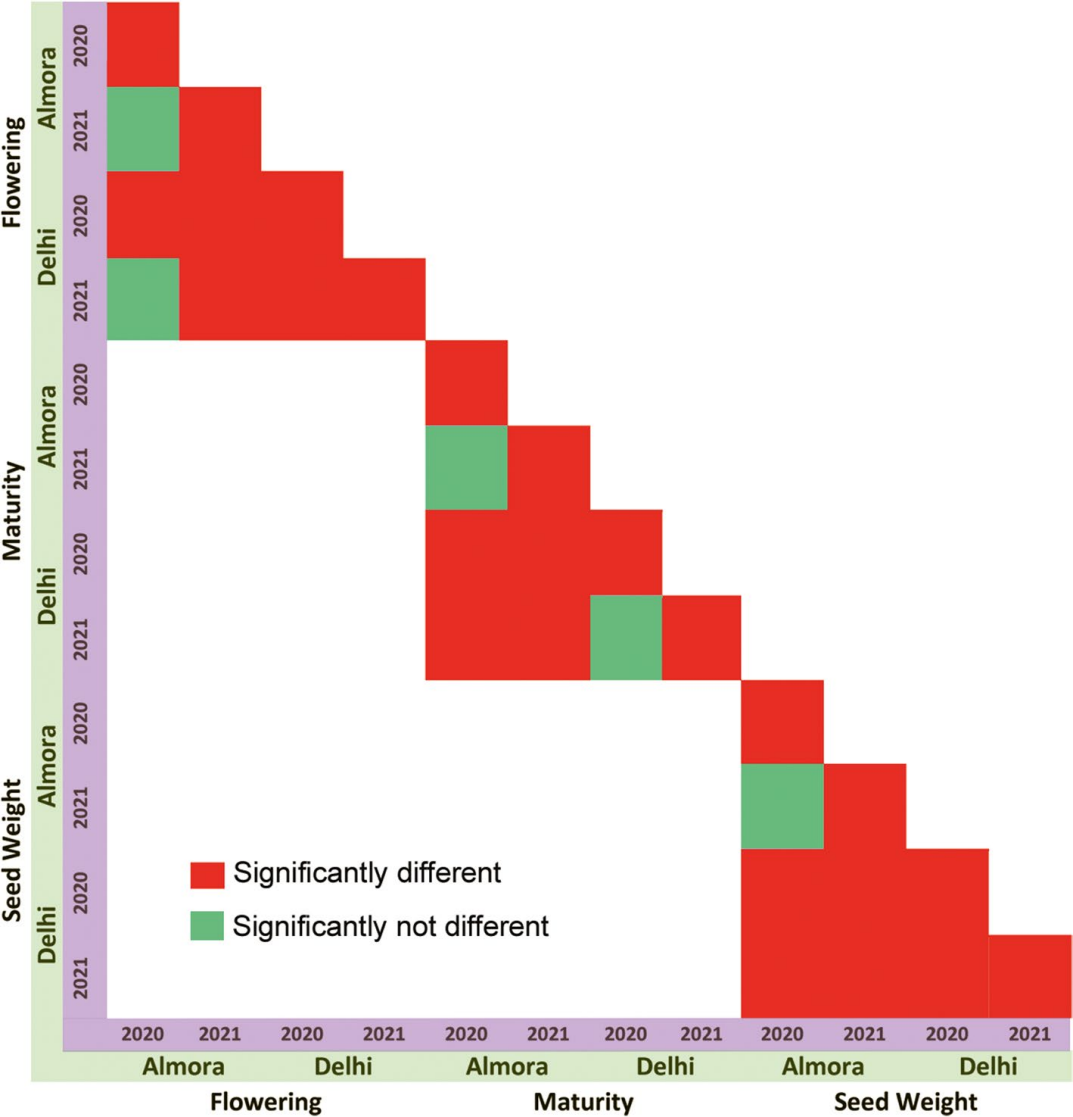
Analysis of significant difference in the phenotypic data of two years (2020–2021) from two locations (Delhi and Almora) based on ANOVA

**Fig. 3:**
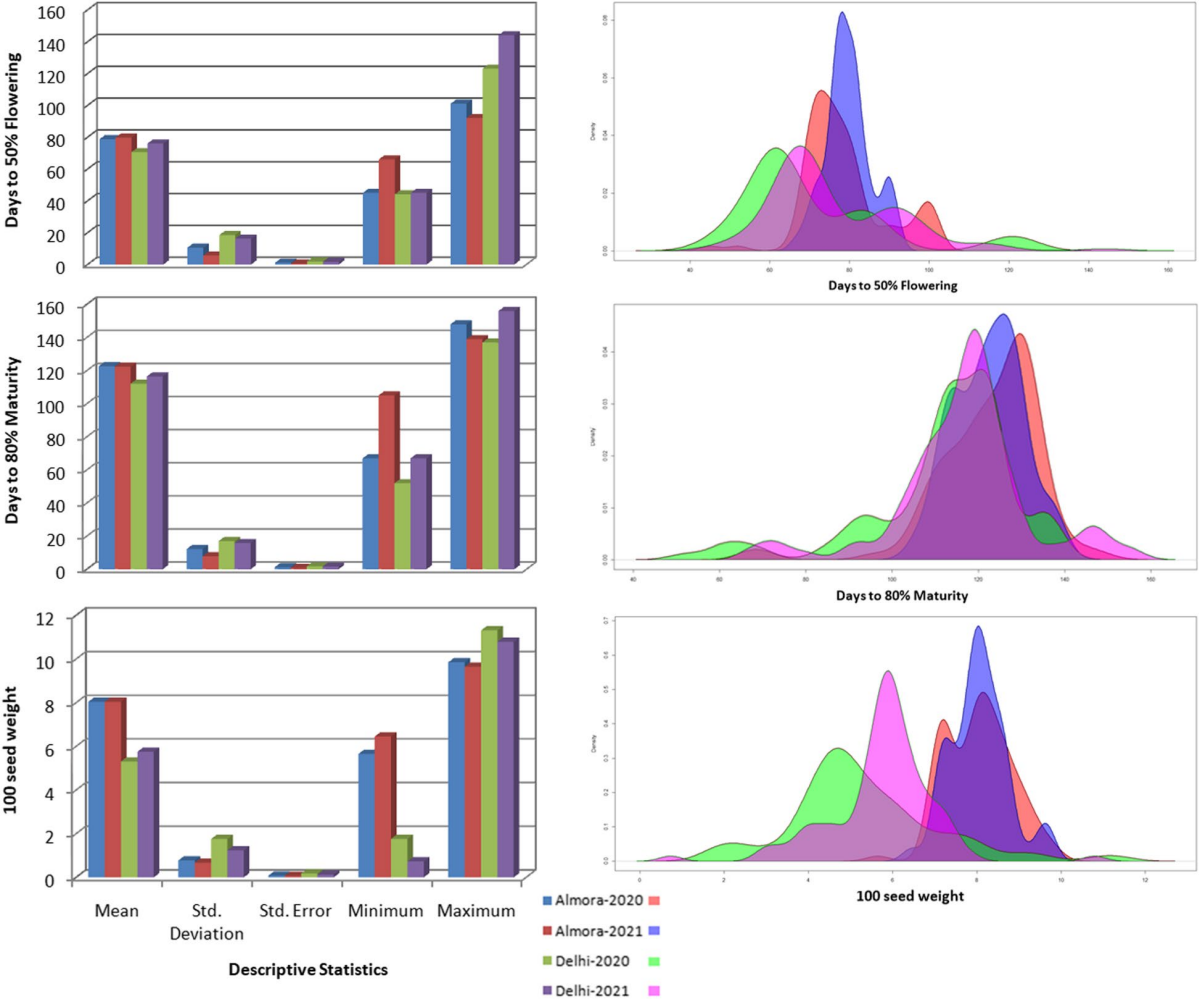
The distribution of phenotypic data under each trait for all the datasets showing the descriptive statistics and density plots

The phenotypic data for two years (2020 and 2021) from two locations (Delhi and Almora) for 100 selected accessions for some traits were found to be significantly different from each other either location-wise or year-wise (Fig. 2). The distribution of trait data for all the datasets is shown in Fig. 3. The minimum, maximum and mean values of Almora datasets were found within a short range (days to 50% flowering: 45–101 days, days to 80% maturity: 67–148 days, 100-seed weight: 5.65–9.68 g), whereas in case of Delhi datasets, these values are observed within a long range (days to 50% flowering: 44–144 days, days to 80% maturity: 52–156 days, 100-seed weight: 0.73–10.79 g) probably due to unexpectedly changing climatic conditions at Delhi. Further, the density plot for days to 50% flowering reveals similar distribution of Delhi datasets, whereas the density plot for 100-seed weight shows similar distribution of Almora datasets. However, for maturity data points of all the datasets were found to be homogeneously distributed. Furthermore, two accessions; EC934417 and IC116118 were identified as early flowering (50 days and 65 days to 50% flowering respectively, averaged over 4 locations). Additionally, these accessions also exhibit early maturation, with EC934417 taking 77 days and IC116118 taking 98 days on an average to reach 80% maturity across the same four locations.

### Processed genotypic data

The initial filtration of sequencing reads before variant calling generated cleaned short reads. The number of bases and reads before and after filtration is given in Table S2. The pre-processing of genotypic data with marker filtration parameters like indels, non bi-allelic markers, minor SNP states, minor allele frequency (< 5%), missing genotype (> 20%), heterozygosity (< 50%) resulted in 49,271 markers. Further, the implementation of genotype filtration parameters retained all the genotypes as all have missing data < 30% and heterozygosity < 50%. The final processed genotypic data contained 49,271 markers for 100 genotypes, for which the phenotypic data contained observations for 50% flowering, 80% maturity and 100 seed weight.

### Population structure

**Fig. 4:**
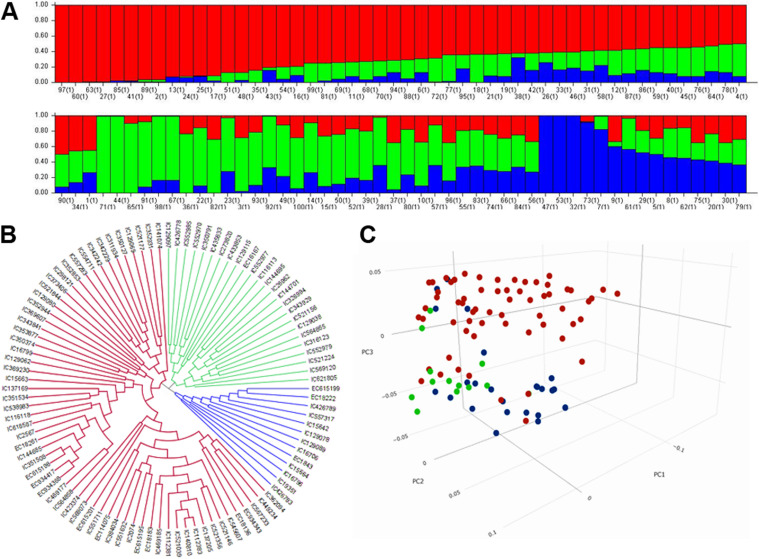
Three putative sub-populations revealed from (**A**) the STRUCTRE-MCMC simulations, (**B**) the clustering based on Neighbor Joining method and (**C**) the plot of first three principal components

The calculated GD values for each marker varied from 0.0582 to 0.5000 with an average of 0.2690 whereas the PIC values varied from 0.0565 to 0.3750 with an average of 0.2253 (Figure S1). The markers having the PIC above 0.3500 were found to have the GD above 0.4500. Therefore, the STRUCTRE software was executed with 5416 markers having high PIC and GD that is expected to correctly determine the number of populations. The best value of K with the highest ∆K was determined to be 3 (Figure S2) suggesting that the genotypes are distributed in three putative populations (Fig. 4A). Further, three putative populations have also been revealed through the genotypic cluster (Fig. 4B) by Neighbour Joining method and by plotting the first three principal components (Fig. 4C). Though the genotypic clustering reveals 3 distinct populations split from the root node, STRUCTRE showed a few admixed individuals in three sub-populations. Large number of pure individuals was observed in the largest cluster (sub-population-1 with 53 individuals), followed by a smaller cluster (sub-population-2 with 32 individuals) than sub-population-1 and the smallest cluster (sub-population-3 with 15 individuals). Although sub-population-2 is larger than sub-population-3, the number of pure individuals in these sub-populations are nearly equal. These three putative sub-populations when matched with the passport information of the cultivars (Table S1), sub-population -1 was found to contain mostly the individuals of eastern and north-eastern regions of India and sub-population -2 was observed to have mostly the north Indian cultivars. The sub-population -3 was noticed to contain mixed individuals. However, few exotic cultivars were also noticed to fall within the sub-populations-1 & 2. The distribution of Indian cultivars is given in the Fig. 1.

### LD decay

**Fig. 5:**
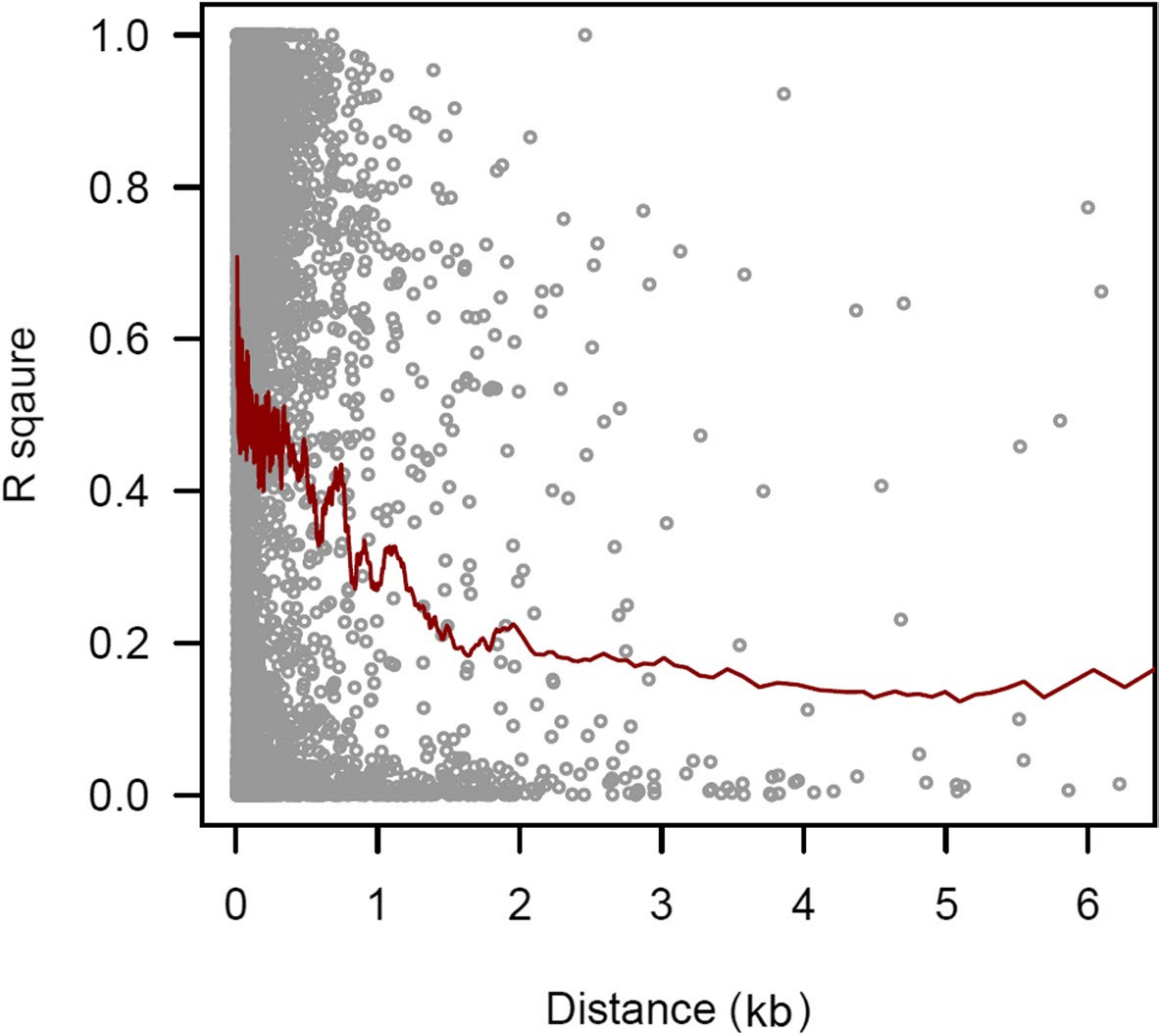
The decay in LD plotted by estimating the LD between all possible pair-wise markers. The LD decays at a distance of 1.5 kb at the *R*-square cutoff of 2%

The LD was observed to be decayed at a distance of 1.5 kb at the r^2^ cutoff of 2% (Fig. 5). Further, the r^2^ was noticed to be on an average of 5% within a distance of 0.5 kb; however, it decayed rapidly from 4 to 2% within a distance of 0.5 kb to 2 kb. After that, the trend line seems to be in equilibrium up to a distance of 6 kb.

### Marker-trait association

#### Marker-trait association with Almora datasets

**Fig. 6:**
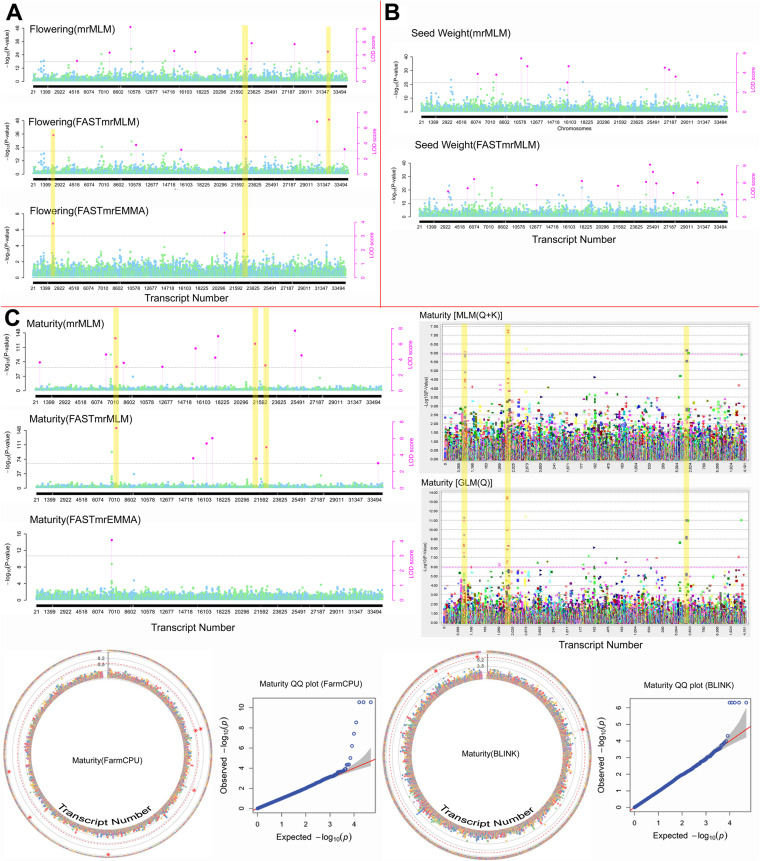
Significantly associated markers for (**A**) flowering, (**B**) seed weight and (**C**) maturity shown on the Manhattan plots, predicted by various models using the phenotypic dataset from Almora in 2020. The yellow colour highlighted regions show the consistent markers predicted by at least two models. The Manhattan plots are not generated for the markers that are predicted by ISIS EM-BLASSO

For Almora 2020, a total of 33 markers have been detected by single and multi-locus models for all the considered traits (Table 1, Fig. 6). The markers considered here are predicted by at least two methods. With this dataset, 3 markers for flowering, 24 markers for maturity and 7 markers for seed weight were detected. However, one marker (SVUTC25856_1400) was found common for both maturity and seed weight traits. The marker, SVUTC06910_1648, predicted for maturity was predicted by 6 out of 10 GWAS models. Further, five markers at different positions (177 bp, 1616 bp, 1648 bp, 1702 bp and 1746 bp) were found significant on one transcript VUTC06910, which are predicted to be associated with maturity trait. The remaining markers in this dataset were predicted on different transcripts.

**Table 1:** Associated markers for flowering, maturity and seed weight predicted using phenotypic data of Almora location in the year 2020

**Marker ID**^a^	**SNP**^c^	**Transcript ID**	**Position (in bp)**	**Models**^b^	**Trait(s)**	**-log**_**10**_** (P)/ LOD**^c^	**MAF**^c^	**R**^**2**^
SVUTC32100_1612	C/T	VUTC32100	1612	6,8,5	Flowering	*LOD*: 4.52 to 7.07	0.260	5.63 to 11.99
SVUTC02300_1585	C/A	VUTC02300	1585	6,7	Flowering	*LOD:* 6.15 to 11.00	0.464	7.10 to 5.38
SVUTC22319_438	C/T	VUTC22319	438	6,7	Flowering	*LOD:* 3.84 to 7.72	0.355	4.72 to 9.66
SVUTC06910_1648	C/T	VUTC06910	1648	4,3,2,1,9,10	Maturity	-*log10(P):* 6.31 to 13.33	0.055	38 to 47
SVUTC06910_1702	G/T	VUTC06910	1702	4, 3,2,1,10	Maturity	-*log*_*10*_*(P):* 6.31 to 13.33	0.055	38 to 47
SVUTC06910_1746	C/G	VUTC06910	1746	4,3,2,1,10	Maturity	-*log10(P):* 6.31 to 13.33	0.055	38 to 47
SVUTC06910_177	C/G	VUTC06910	177	4,3,2,1	Maturity	-*log10(P):* 6.79 to 13.51	0.063	37 to 49
SVUTC02283_1297	G/T	VUTC02283	1297	4,2,1	Maturity	-*log10(P):* 6.07 to 10.96	0.092	28 to 41
SVUTC07229_692	T/C	VUTC07229	692	6,5,8	Maturity	*LOD*: 5.68 to 7.24	0.220	23 to 24
SVUTC08788_874	A/G	VUTC08788	874	3,2,1	Maturity	*-log10(P):* 6.20 to 11.35	0.057	29 to 43
SVUTC21395_469	A/G	VUTC21395	469	5,8,6	Maturity	*LOD:* 3.56 to 6.03	0.200	8.33 to 18.24
SVUTC27187_1237	C/T	VUTC27187	1237	3,2,1	Maturity	*LOD*: 6.15 to 11.00	0.060	30 to 43
SVUTC27441_1674	A/C	VUTC27441	1674	3,2,1	Maturity	*-log10(P):* 5.99 to 10.97	0.060	28 to 41
SVUTC06910_1616	C/G	VUTC06910	1616	7,1	Maturity	*-log10(P):* 6.61 *LOD:* 4.07	0.056	10 to 27
SVUTC16925_1108	T/C	VUTC16925	1108	8,6	Maturity	*LOD*: 6.02 to 7.39	0.262	13.33 to 14.64
SVUTC31321_227	A/C	VUTC31321	227	8,10	Maturity	*log10(P):* 6.30 *LOD:* 5.43	0.100	16.84
SVUTC34182_2284	C/T	VUTC34182	2284	6,10	Maturity	*log10(P):* 6.31 *LOD:* 3.01	0.306	4.60
SVUTC25856_1400	T/G	VUTC25856	1400	5,8,6	Maturity, Seed weight	*LOD:* 3.99 to 5.88	0.214	7.06 to 13.22
SVUTC06074_3949	G/A	VUTC06074	3949	8,6	Seed weight	*LOD:* 3.85 to 6.63	0.146	9.59 to 11.35
SVUTC12822_2877	A/G	VUTC12822	2877	8,6	Seed weight	*LOD:* 4.36 to 5.59	0.191	6.99 to 7.43
SVUTC21543_375	C/T	VUTC21543	375	8,6	Seed weight	*LOD*: 3.5 to 5.46	0.247	8.00 to 11.45
SVUTC25312_5309	G/A	VUTC25312	5309	8,6	Seed weight	*LOD:* 9.18 to 9.86	0.468	6.90 to 12.33
SVUTC25491_6046	T/C	VUTC25491	6046	8,6	Seed weight	*LOD*: 7.91 to 8.72	0.261	3.15 to 4.06
SVUTC30884_3008	T/G	VUTC30884	3008	8,6	Seed weight	*LOD:* 6.03 to 11.58	0.205	10.51 to 26.26

^a^The markers highlighted in yellow are associated with more than one trait

^b^1. GLM (Q), 2. GLM (PCA)], 3. MLM (Q + K), 4. MLM (PCA + K), 5. mrMLM, 6. FASTmrMLM, 7. FASTmrEMMA, 8. ISIS EMBLASSO, 9. FarmCPU, 10. BLINK

^c^*SNP* single nucleotide polymorphism, *LOD* logarithm of the odds, *MAF* minor allele frequency

In the case of phenotypic data collected from the Almora location in the year 2021, a total of 11 markers were detected to be associated with different traits (Table S3, Figure S3). For this dataset, only multi-locus models of mrMLM have detected significant markers which are selected based on the LOD score. In this case, 3 markers (SVUTC21295_283, SVUTC22319_77, SVUTC31327_3358) for flowering, 3 markers (SVUTC21295_283, SVUTC05378_2089, SVUTC21831_6083) for maturity and 6 markers (SVUTC01132_259, SVUTC06850_2182, SVUTC09951_1960, SVUTC23058_1028, SVUTC24042_2561, SVUTC24699_1593) for seed weight traits were predicted where one marker (SVUTC21295_283) was found common for both flowering and maturity traits. Here, all the markers except SVUTC21295_283 were predicted by exactly two different multi-locus models. Though SVUTC21295_283 has been predicted by only one method, it was considered, as it was predicted for two different traits. All the markers were predicted on different transcripts. However, one transcript VUTC22319 was found common in both years with different marker positions (438 in 2020 and 77 in 2021) and was found to be associated with flowering for the datasets of both years.

#### Marker-trait association with Delhi datasets

The marker-trait association analysis for phenotypic data of Delhi in the year 2020 revealed a total of 58 markers, out of which the majority of the markers (49) were for flowering trait, whereas 4 for maturity and 5 for seed weight were identified (Table S4, Figure S4). Here, none of the markers were found to be associated with more than one trait. The 58 markers are located on 24 transcripts, where seven transcripts contain more than two markers each. The transcript VUTC28154 contains the highest (8) number of markers on it. Out of 49 markers of flowering, 43 markers were predicted only by single locus GWAS models. A marker, SVUTC28154_1646 predicted to be associated with flowering, has been predicted by 8 out of 10 considered GWAS models.

For the phenotypic data collected from Delhi in 2021, the GWAS analysis revealed the association of 49 markers for the three considered traits (Table S5, Figure S5). Out of these 49 markers, 4 for seed weight, 4 were for maturity and 42 were found to be associated with flowering. Interestingly, one marker (SVUTC25248_8323) being predicted by three methods was found to be associated with maturity and seed weight. Nine transcripts associated with flowering were identified by at least two markers on them, where the transcript VUTC28201 contains highest number of markers on it. A total of 15 markers and eight transcripts were found common between two years of data of Delhi, all of which were observed to be associated with the flowering trait.

### Associated markers and corresponding transcripts

**Fig. 7:**
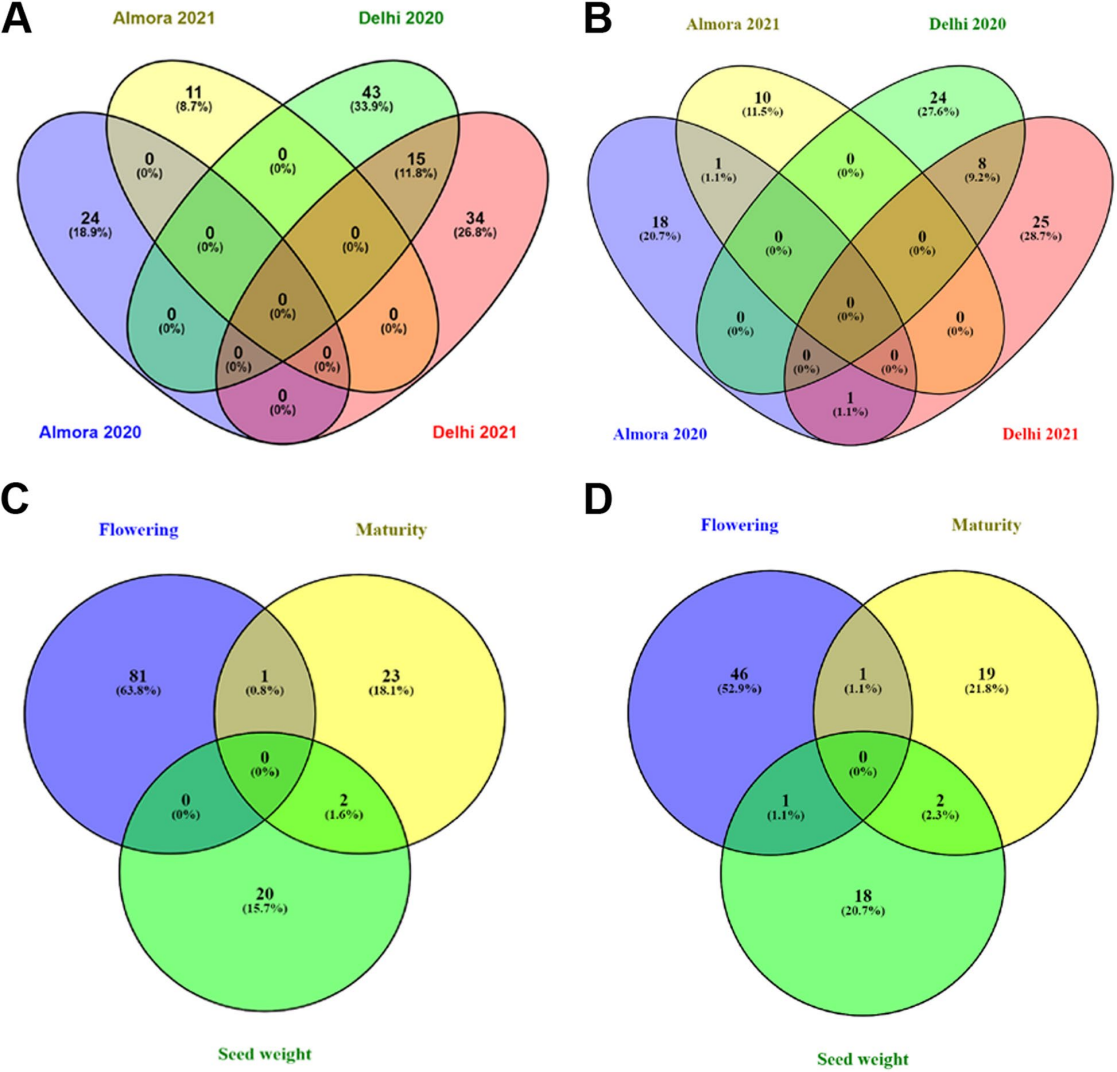
Common markers and transcripts identified for different traits and different datasets. (**A**) Common markers for datasets, (**B**) common transcripts for datasets, (**C**) common markers for traits, (**D**) common transcripts for traits

From the overall analysis, 87 transcripts were found associated with the traits, having 127 markers in total from all the datasets considered. From the datasets of two locations in two consecutive years, neither any marker nor any transcript could be found in common for all. However, 15 markers were noticed to be common between Delhi-2020 & Delhi-2021 datasets (Fig. 7A). Transcripts are concerned, one transcript (VUTC22319: flowering) between Almora-2020 & Almora-2021 datasets and 8 transcripts (VUTC28154, VUTC28165, VUTC28183, VUTC28185, VUTC28192, VUTC28201, VUTC29109, VUTC28186; all associated with the flowering trait) between Delhi-2020 and Delhi-2021 datasets were identified to be common (Fig. 7B). Interestingly, one transcript (VUTC25312: flowering) was also found common between Almora-2020 and Delhi-2021 datasets, even though no marker was identified to be common between these two datasets.

From 3 out of 127 markers and 4 out of 87 transcripts were identified to be associated with more than one trait. One marker (SVUTC21295_283) between flowering and maturity and two markers (SVUTC25856_1400, SVUTC25248_8323) between flowering and seed weight were identified (Fig. 7C). Further, one transcript (VUTC21295) between flowering and maturity, two transcripts (VUTC25856, VUTC25248) between maturity and seed weight and one transcript between flowering and seed weight (VUTC25312) have been identified (Fig. 7D). However, later one was predicted for two different datasets (Almora-2020 and Delhi-2021 with two different markers *i.e.,* SVUTC25312_5309 and SVUTC25312_4994, respectively). These common markers and transcripts are expected to help understand the interrelation between the considered traits in terms of the governing genes.

### Annotation of candidate genes using associated transcript sequences

**Fig. 8:**
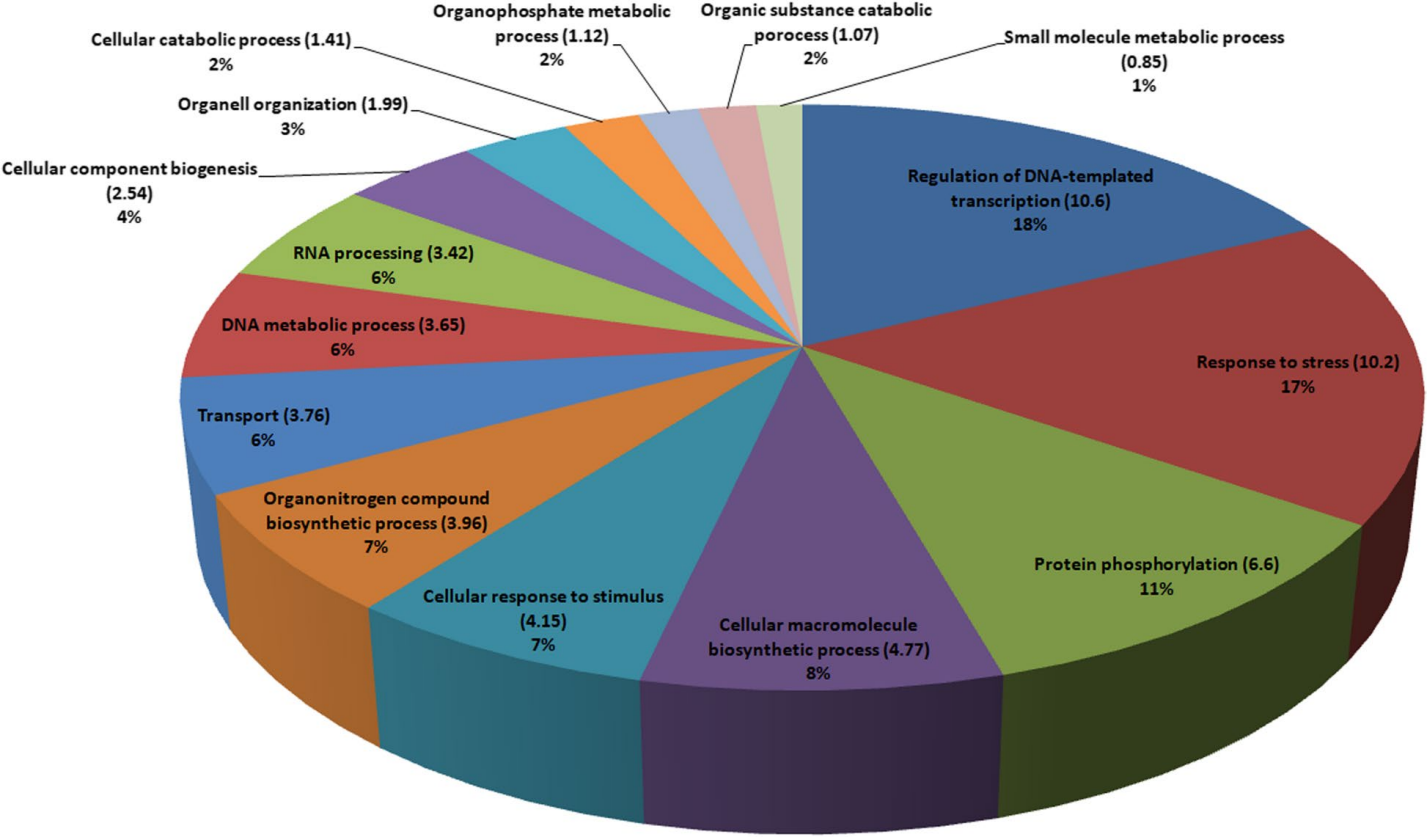
The major gene ontology terms for biological processes obtained as annotations for 87 transcripts

The annotation of transcripts in terms of chromosomal localization (Table S6) and molecular function (Table S7) has revealed many functional proteins related to the associated traits. Based on the BLAST results, majority of transcripts showed top hits against the proteins of *V. umbellata* and *Vigna anguilaris.* BLAST2GO revealed GO terms for each transcript sequence. The major GO terms for biological processes obtained as annotations for 87 transcripts are presented in the form of a pi-chart in Fig. 8. From the annotation of candidate genes, the potential contributors to the process of flowering, such as HSC80, P-II PsbX, phospholipid-transporting-ATPase-9, pectin-acetylesterase-8, and E3-ubiquitin-protein-ligase-RHG1A have been identified. Additionally, two genes, WRKY1 and DEAD-box-RH27 were found to be associated with only seed weight, while the association of PIF3 [having Basic Helix-Loop-Helix (bHLH) motif] and pentatricopeptide-repeat-containing-gene with maturity and seed weight and aldo–keto-reductase with flowering and maturity were identified.

### Chromosomal localization of markers

**Fig. 9:**
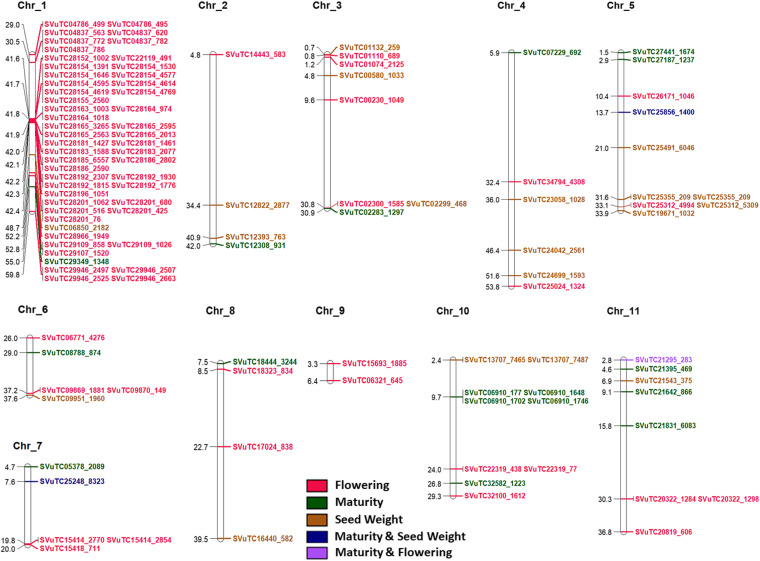
The trait-wise associated markers mapped onto different chromosomes of the rice bean cultivar FF25

The exact chromosomal positions of the associated markers were revealed from the ungapped alignment between marker sequences (101 bp; SNP at 51st position) and chromosomal sequences of the recently released rice bean cultivar FF25. The trait-wise associated markers mapped onto different chromosomes of rice bean is shown in Fig. 9. The associated markers were found distributed over all the chromosomes of rice bean. Most of the markers associated with flowering traits are found on chromosome 1. The highest number of markers for maturity was found on chromosome 11, whereas the highest number of markers for seed weight was found on chromosome 5. On chromosome 9, only two markers for flowering were identified. A set of 35 markers were identified on chromosome 1 within a distance of 80.83 kb at 41.6 Mbp to 42.4 Mbp.

### Inter-species synteny based on chromosomal localization of associated transcripts

**Fig. 10:**
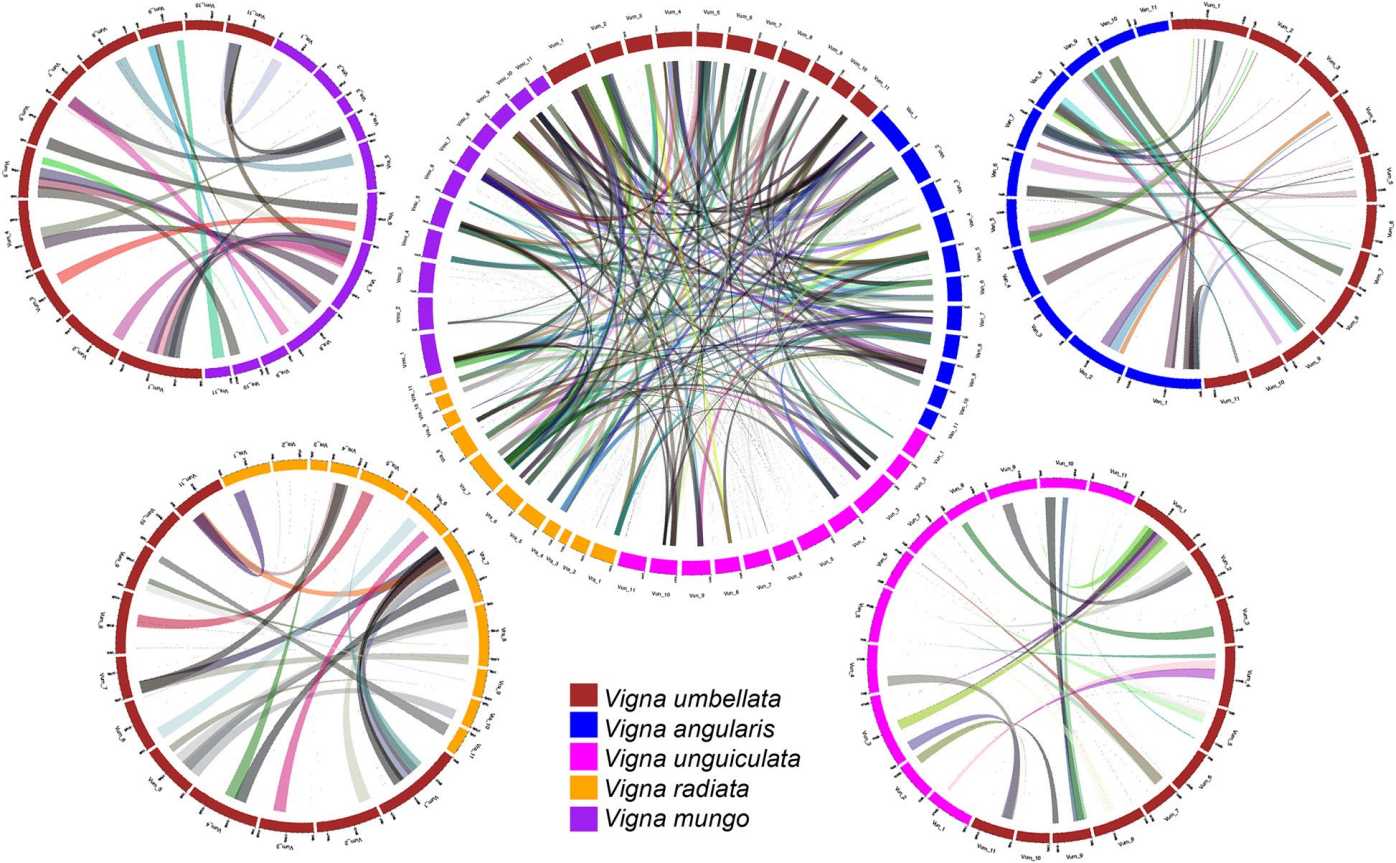
Synteny between all the considered *Vigna* genomes in terms of the trait-associated transcripts along with pair-wise synteny between *V. umbellata* and other *Vigna* species

The associated transcripts, when mapped onto the recently released genome of rice bean [13]; with >  = 95% query coverage, all the associated transcripts were mapped onto the chromosomes (Table S6). 81 out of 87 transcripts were identified with 100% percentage identity and >  = 96% query coverage. Further, the mapping of the 87 transcripts onto the chromosomes of different *Vigna* species revealed their inter-species chromosomal locations, which assisted in establishing a synteny between all the considered *Vigna* genomes in terms of the trait-associated transcripts (Fig. 10). The pairwise synteny between *V. umbellata* and other *Vigna* species revealed the inter-species chromosomal syntenic relationship. Based on these pair-wise relationships with *V. umbellata,* the synteny between other *Vigna* species was also established (Fig. 10).

### Expression analysis of associated transcripts

**Fig. 11:**
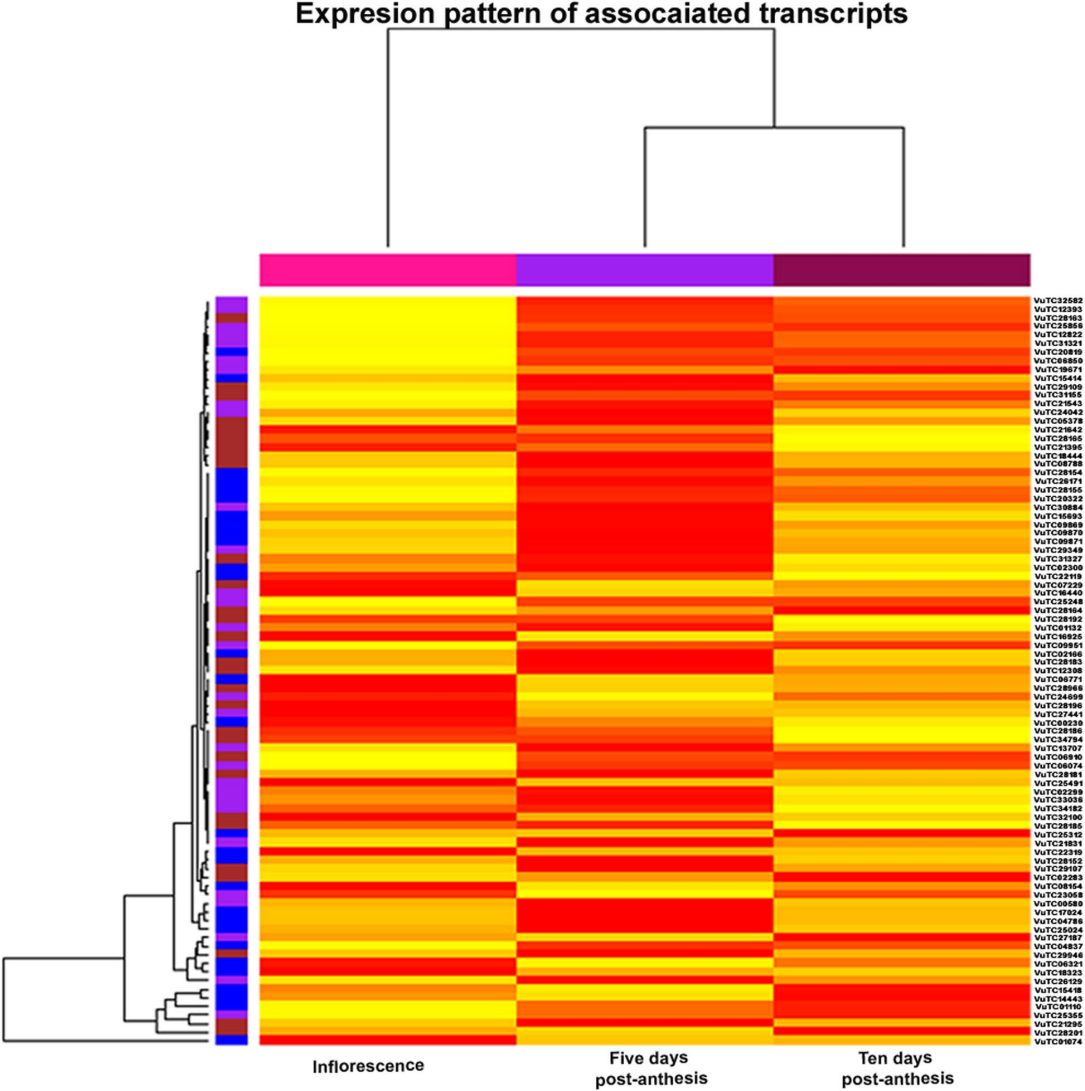
The expression pattern of genes represented by 87 transcripts for the stages like inflorescence, 5 days post anthesis and 10 days post anthesis

The expression pattern of genes represented by 87 transcripts for the stages like inflorescence, 5 days post anthesis and 10 days post anthesis is given in the Fig. 11. Most of the associated transcripts for flowering were found enriched with the reads from samples taken at inflorescence and five days post anthesis stages. However, the few transcripts associated with maturity and seed weight were also observed to be enriched with the reads from all three stages, suggesting their expression during the entire process of flowering to maturity. Among the top 10 expressed transcripts across all three stages, five transcripts associated with flowering were found to encode the proteins such as heat shock cognate protein 80 (HSC80), Photosystem II PsbX (P-II PsbX), plasma membrane ATPase 4, photosystem II stability/assembly factor HCF136, and 40S ribosomal protein S19-1.

## Discussion

Rice bean, being an underutilized crop with high nutritional value, bears the potential to contribute to the food and nutritional security across the globe [1, 7, 8]. However, its importance is being recognized, and several research projects have been undertaken for the genetic improvement of this crop. The genomes released by the Chinese Academy of Agricultural Sciences, Beijing, China [13] at the chromosomal level and the International Centre for Genetic Engineering and Biotechnology, New Delhi, India, at the scaffold level [15] have increased the scope of genomic research on rice bean. The phenotypic data for 100 rice bean varieties considered here, collected from two locations in two consecutive years, was found significantly different for most of the traits (Fig. 2). The significant difference in the phenotypic data is expected for different locations as Almora has a hilly topography and receives high rain, whereas Delhi has a plain topography and has much less rain during the crop season. However, the difference in the data during consecutive years in the same location could be due to unpredictable climate change at these two locations in consecutive years.

Information on population structure in GWAS analysis is often incorporated to avoid false marker-trait associations. Q-matrix generated through STRUCTURE and PC scores from PCA help infer the population structure from the genotypic data in GWAS analysis [46]. STRUCTURE and PCA indicated the classification of genotypes into three subpopulations (sub-population 1–3) that have also corroborated with the genotypic cluster derived through TASSEL (Fig. 4). Sub-population 1 contains 53 genotypes with 80 days on an average to 50% flowering, 123 days on an average to 80% maturity and an average 100-seed-weight of 6.96 g (Table S8). Further, sub-population 2 was noticed to have 32 genotypes with average values of 74 days, 116 days, and 6.61g for 50% flowering, 80% maturity and 100 seed weight, respectively (Table S8). Furthermore, sub-population 3 was observed to have a group of 15 genotypes with average values of 69 days, 106 days and 6.54g for 50% flowering, 80% maturity and 100-seed-weight, respectively (Table S8). This implies that sub-population 1 contains mostly the late flowering and late maturity genotypes, whereas sub-populations 2 and 3 contain most of the genotypes of early and normal flowering types.

In cross pollinated crops LD decays at short distances as compared to self-pollinated crops [47, 48]. Rice bean is a highly cross-pollinated crop, thus, we have also observed decay of LD at short distances. The LD decay at an *r*^2^ cutoff of 2% [49, 50] was observed to be 1.5 Kb (Fig. 5), suggesting a high genetic diversity in the genotypes taken into consideration. Further, the LD decay at short distances can also be expected with the transcript data as mature mRNA lacks introns. Thus, markers identified through GWAS within a distance of 1.5 kb are expected to be associated with similar or related traits and are expected to be inherited together with a little chance of contemporary recombination. Several GWAS models exist with their own advantages and disadvantages. We implemented ten different models and selected markers on the basis of their threshold scores. The markers were further screened if they are predicted by at least two models. Our approach was to minimize the false positive as well as not to discard biologically important markers even if they were not predicted by high number of methods.

In spite of applying several models, we could not find any marker for any trait found consistently with all datasets. However, we observed few markers and corresponding transcripts consistent with the two datasets (Fig. 7). It can be expected as that two locations included in our study, Almora and Delhi differs in their topography, latitude, longitude and climate conditions, causing large differences in the phenology and related traits and thus necessitating the involvement of different set of genes at these two locations. This assertion is supported by the findings of Guan et al*.* [13] who reported distinct association signals for the different locations. They conducted GWAS analysis for various traits including *i.e.,* flowering and seed weight, which were considered in our study. Two of their reported markers, Chr6: 25.86 Mb and Chr10: 28.15 Mb have shown association with flowering. We have also observed the association of two markers (SVUTC06771_4276: Chr6: 26.03 Mb and SVUTC32100_1612: Chr10: 29.31 Mb, respectively) for flowering within the vicinity (1.5 Mb) of their reported markers. Additionally, we found four markers (SVUTC06850_2182: Chr1: 48.73 Mb, SVUTC25856_1400: Chr5: 13: 66 Mb, SVUTC09951_1960: Chr6: 37.65 Mb, SVUTC25248_8323: Chr7: 7.58 Mb) for seed weight nearby the four markers (Chr1: 48.26 Mb, Chr5: 14.78 Mb, Chr6: 36.35 Mb, Chr7: 6.14 Mb, respectively) reported by Guan et al. [13] for seed weight. However, two of these markers (SVUTC25856_1400: Chr5: 13.66 Mb, SVUTC25248_8323: Chr7: 7.58 Mb) identified in our study were also found to be associated with maturity trait.

The chromosomal localization of the genes revealed the flowering genes on chromosome 1 (Fig. 9), and the role of chromosome 1 in controlling the flowering time has been reported in common bean [51–53]. It is worth mentioning here that chromosome 1 of cow pea corresponds to chromosome 1 of common bean [54] and chromosome 1 of rice bean [13], which indicates the correspondence between the common bean and rice bean genome in terms of chromosome 1. The inter-species syntenic relationship (Fig. 10) based on associated transcripts revealed the higher closeness of rice bean with *V. angularis*, *V. mungo* and *V. radiata* than *V. unguiculata*. The same has also been depicted in Guan et al. [13] and Pattanayak et al. [7].

Among the identified markers, SVUTC25856_1400 associated with maturity and seed weight traits located on the transcript (VUTC25856) that has shown similarity with transcription factors bHLH149, bHLH147 and PIF3. The bHLH transcription factors regulate the expression of the genes related to biosynthesis, metabolism and transduction of plant hormones [55]. The phytochrome-interacting factor (PIF3) belongs to bHLH family of proteins that has a specific function in light-induced developmental processes [56]. During seed maturation in light conditions, the amount of abscisic acid in seeds and sensitivity of seeds to abscisic acid is influenced [57] that has an important role in the biosynthesis of storage compounds in the embryo, seed dormancy, and the inhibition of precocious germination [58–60]. Besides, [61] reported that mutants of *Arabidopsis* deficient with abscisic acid produced seeds with increased size, mass, and embryo cell number. Thus, transcript (VUTC25856) similar to PIF3 with bHLH domain is expected to regulate the abscisic acid-dependent behavior of seeds during light-induced developmental processes.

A transcript VUTC21295 containing the marker SVUTC21295_283 associated with maturity and flowering was found to have aldo–keto reductase function. The aldo–keto reductase enzymes reduce carbonyl substrates like sugar aldehydes, keto-steroids and keto-prostaglandins [62]. The D-GalUA reductase, an aldo–keto reductase, plays an important role in D-Galacturonic acid pathway for ascorbate biosynthesis in plants [63]. Kotchoni et al. [64] reported that an artificial increase in ascorbic acid delayed the flowering. Delaying in flowering affects the days to maturity. Thus, the transcript VUTC21295 having an aldo–keto reductase function might be playing a regulatory role in ascorbate biosynthesis in rice bean, having an effect on its flowering and maturity.

On the transcript VUTC28154, 8 markers were identified where the marker SVUTC28154_1646 was predicted by 8 out of 10 models. The transcript showed similarity with putative phospholipid-transporting ATPase 9, and the GO annotation revealed its involvement in the phospholipid translocation process. Zhou et al. [65] reported that *phospholipid-transporting ATPase* 9, along with other phospholipases, plays a part in accelerating the pollen tube aging in *Pyrus bretschneideri*. Five markers were identified on the transcript VUTC28201 for flowering. The transcript annotation shows its similarity with pectin acetylesterase 8-like protein having a role in cell wall organization and pectin acetylesterase activity. However, the expression of pectin acetylesterase 8 of *Arabodopsis thaliana* has been reported to be highly regulated during plant growth and development. It is expressed in different flowering parts and stages (pollen, inflorescence meristem, stamen, petal differentiation and expansion stage), suggesting a role in the control of the degree of acetylation of pectins [66, 67].

Among the top 10 expressed transcripts in all three stages (inflorescence, 5 days post anthesis and 10 days post anthesis), 8 transcripts are found common. Out of these eight transcripts, associations of 5 transcripts with flowering, one with both flowering and maturity, one with maturity, and one with seed weight have been revealed. The transcripts associated with only flowering were found to encode heat shock cognate protein 80 (HSC80; VuTC01074), Photosystem II PsbX (P-II PsbX; VuTC14443), plasma membrane ATPase 4 (VuTC29946), photosystem II stability/assembly factor HCF136 (VuTC01110) and 40S ribosomal protein S19-1(VuTC15418). The strong expression of HSC80 in floral shoot apices until six days post anthesis has been observed by Koning et al. [68]. Further, the expression of P-II PsbX [69] and plasma membrane ATPase 4 [70] in the flowers of *Spinacia oleracea* and *A. thaliana,* respectively has also been reported. Meurer et al., [71] have demonstrated that the *A. thaliana* plants with mutant HCF136 deficient in PSII activity were failed to produce flowers. Another transcript annotated as 40S ribosomal protein S19-1, was shown to be differentially expressed by Yan et al., [72] while analyzing the differential expression in anther and stigma of *Eruca sativa* during pre-bloom and after flowering stages. One transcript (VUTC21295) associated with both flowering and maturity, which codes for aldo–keto reductase has been discussed earlier regarding its association with the related traits. Its appearance within the top 10 expressed transcripts in all three stages confirms its association. A transcript VuTC26129 annotated as WD repeat-containing protein 48(WDR48) and associated with maturity appeared within 10 expressed transcripts for all three stages. A WDR48 analog in *A. thaliana* has been demonstrated to interact and activate a deubiquitinase UBP3 [73]. Additionally, plant deubiquitinases are reported to control flowering, embryogenesis, pollen and seed development [74–76]. The transcript VuTC25355 associated with seed weight was annotated as 60S ribosomal protein L18a (RPL18a) and the role of RPL18a in embryo development has been established by Yan et al., [77].

Apart from all the above discussed genes, a few major genes like WRKY transcription factor 1(VuTC01132: seed weight), E3 ubiquitin-protein ligase RHG1A (VuTC02166: flowering), DEAD-box ATP-dependent RNA helicase 27(VuTC12393: seed weight), pentatricopeptide repeat-containing protein (VuTC09951: seed weight, VuTC31321: maturity) were found to contain trait associated markers. A *WRKY1* gene of *Solanum chacoense* has been reported to be involved in the process of seed development, having a specific role during the process of embryogenesis, and was also found to be highly expressed in fertilized ovules at the late torpedo stage in wild potato [78]. Shu and yang, [79] demonstrated the role of E3 ubiquitin-protein ligase RHG1A protein containing RING-H2_TTC3 type domain in flowering time control and light response. Further, the role of deadbox RH27 in the process of seed development in *A. thaliana* has been established by Hou et al. [80] through a mutagenesis experiment. They reported that a recessive mutation in the gene produced shrivelled or wrinkled seed.

The associative transcriptomics approach followed in the present investigation revealed a total of 127 markers on 87 transcripts associated with flowering, maturity and seed weight traits. Besides, 81 markers on 46 transcripts for flowering, 23 markers on 19 transcripts for maturity, 20 markers on 18 transcripts for seed weight, 2 markers on 2 transcripts for both seed weight and maturity, 1 marker on 1 transcript for both flowering and maturity were found associated. However, 1 transcript was found associated with both flowering and seed weight with different markers for both traits. The functional annotation of transcripts revealed the corresponding gene description, domains, super families and GO terms of all the associated transcripts. Further, the role of the identified genes in influencing the corresponding traits has been corroborated with findings in other crops. The association analysis involving SNPs obtained from transcriptome-based variant calling followed in this study is expected to provide insights into genetic mechanisms governing economically important production traits for various crops.

### Supplementary Information

**Supplementary Material 1. **

## Data Availability

The transcriptome data used in this study has been submitted to NCBI under the Bioproject accession PRJNA916051 (https://www.ncbi.nlm.nih.gov/bioproject/?term=PRJNA916051). The RNA sequenced for two different developing seed stages *i.e.,* 5-days post anthesis and 10-days post anthesis of rice bean (accession: IC426787) has been submitted to NCBI under the accessions SRR16122607 (https://www.ncbi.nlm.nih.gov/sra/SRR16122607) and SRR16122602 (https://www.ncbi.nlm.nih.gov/sra/SRR16122602) respectively.
