# Supplementary material for: Transcriptome-wide association mapping provides insights into the genetic basis and candidate genes governing flowering, maturity and seed weight in rice bean (Vigna umbellata)
Source: BMC Plant Biol. 2024 May 8;24:379. doi: 10.1186/s12870-024-04976-y (PMC11077894; doi:10.1186/s12870-024-04976-y)
Supplement: Supplementary file 1 — Supplementary Material 1. [file 12870_2024_4976_MOESM1_ESM.pdf]

Supplementary material

Article title: Transcriptome-wide association mapping provides insights into genetic basis and candidate genes governing flowering, maturity and seed weight in rice bean *(Vigna umbellata*)

Authors: Tanmaya Kumar Sahu, Sachin Kumar Verma, Gayacharan, Nagendra Pratap Singh, Dinesh Chandra Joshi, D. P. Wankhede, Mohar Singh, Rakesh Bhardwaj, Badal Singh, Swarup Kumar Parida, Debasis Chattopadhyay, Gyanendra Pratap Singh, Amit Kumar Singh

Supplementary Figures

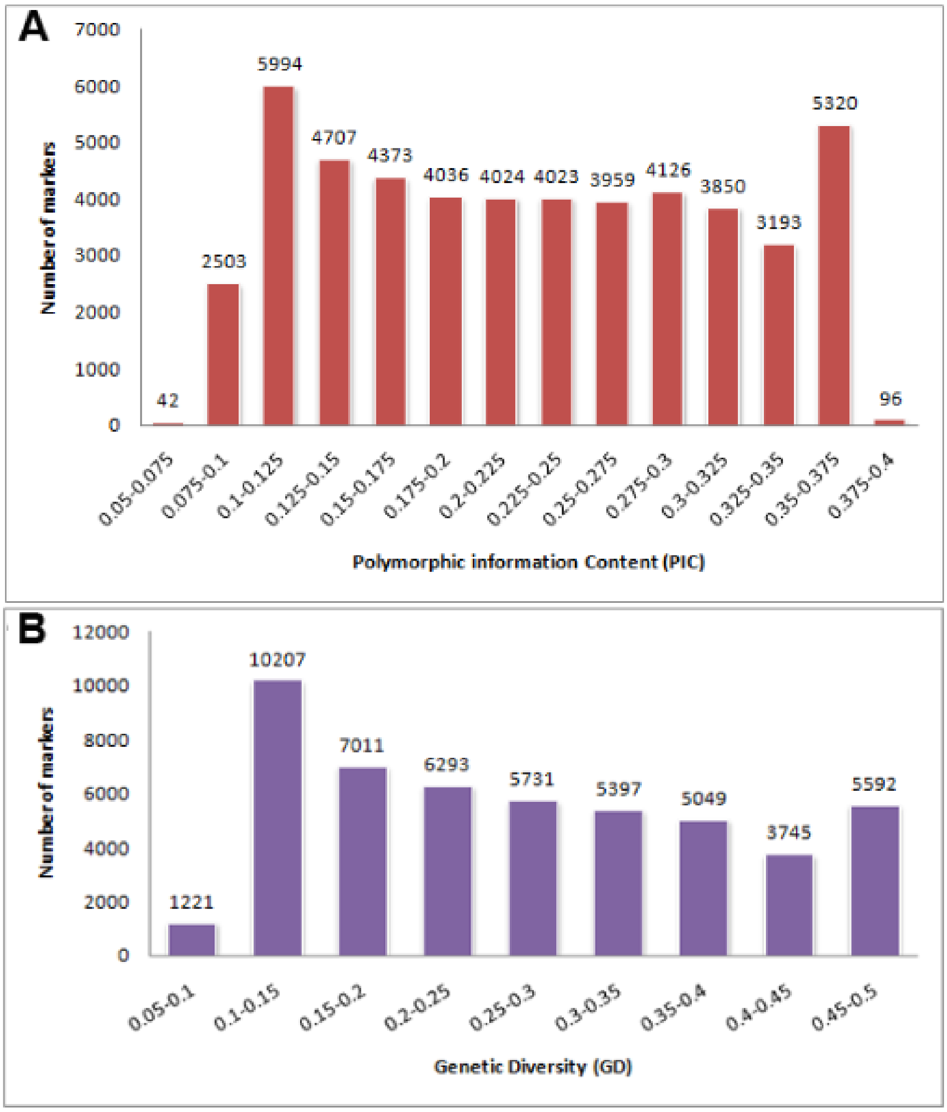

Figure S1. The distribution of (A) polymorphic information content (PIC) and (B) genetic diversity (GD) values calculated from the genotypic data.

(a)

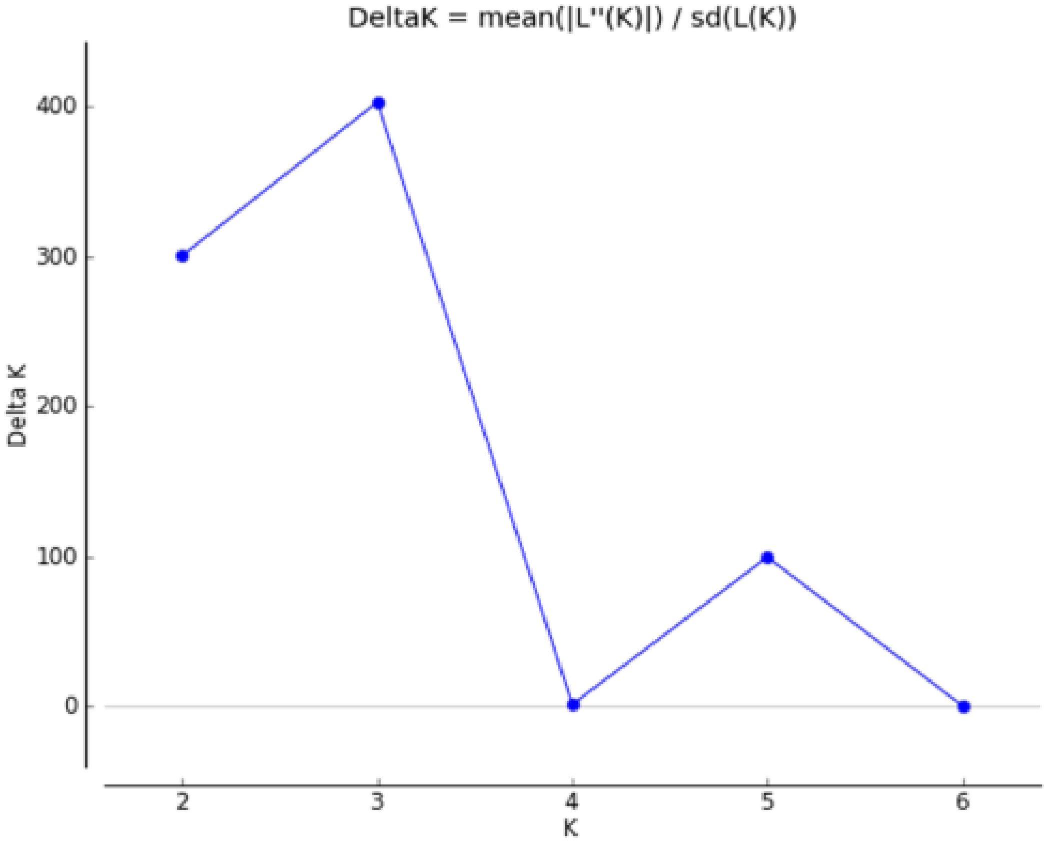

(b)

K	Reps	Mean LnP(K)	Stdev LnP(K)	Ln'(K)	|Ln''(K)|	Delta K
1	10	-682951.800000	12.987088	—	—	—
2	10	-660005.540000	21.355780	22946.260000	6421.970000	300.713439
3	10	-643481.250000	24.932052	16524.290000	10047.550000	402.997313
4	10	-637004.510000	413.665666	6476.740000	647.250000	1.564669
5	10	-631175.020000	1126.960272	5829.490000	112451.570000	99.783083
6	10	-737797.100000	355295.536013	-106622.080000	222497.600000	0.626232
7	10	-621921.580000	1703.562692	115875.520000	—	—

Figure S2. The graph showing the highest value of ΔK at K= 3(*i.e.*, three putative population). (A) Plot showing K in X-axis and ΔK in Y-axis. (B) Values of Mean LnP(K), Standard deviation LnP(K), Ln'(K), |Ln''(K)| and ΔK for all values of K from 1 to 7.

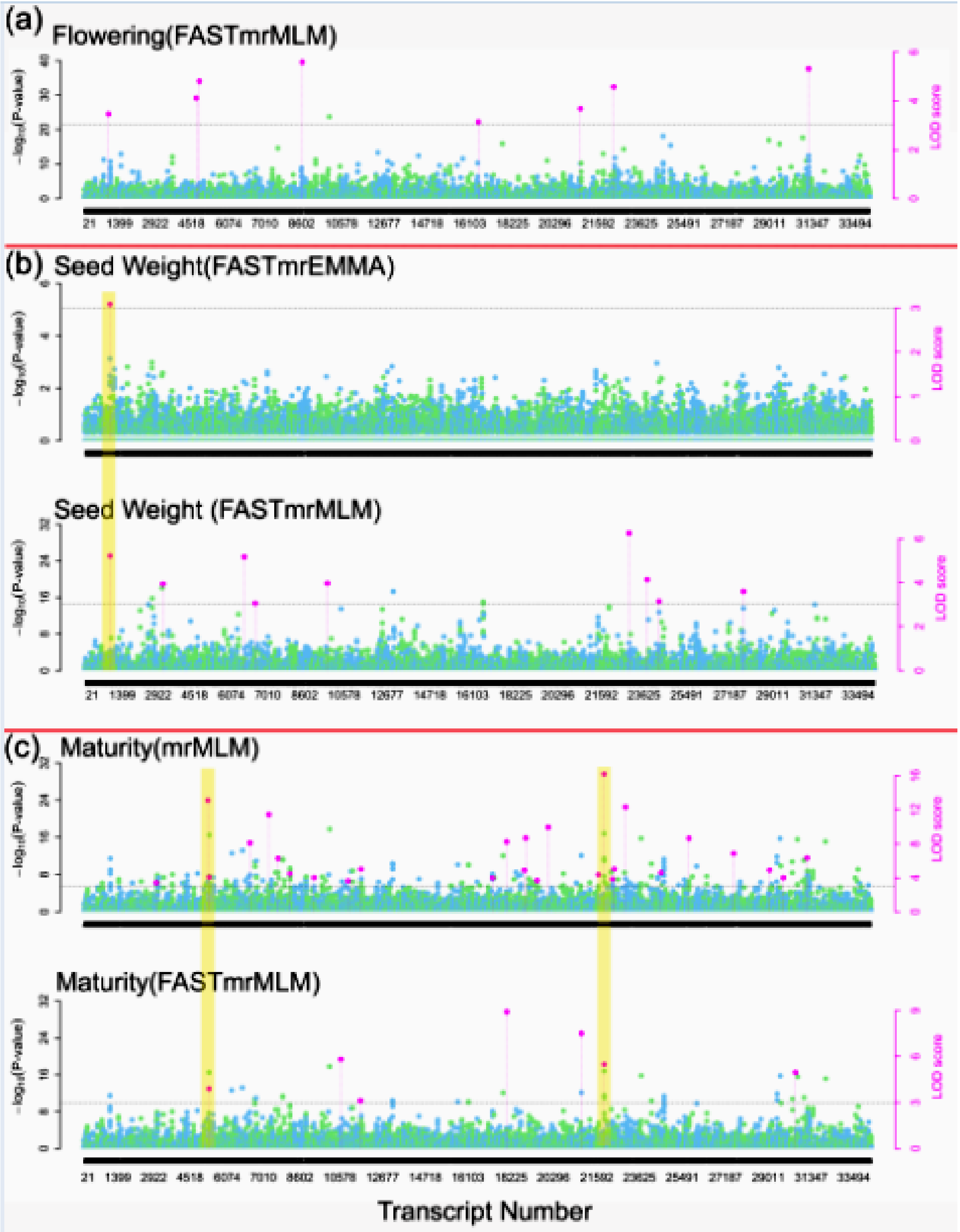

Figure S3. Significantly associated markers for (A) flowering, (B) seed weight and (C) maturity shown on the Manhattan plots, predicted by various models using the phenotypic dataset collected from Almora in the year 2021. The highlighted regions show the consistent markers predicted by at least two models. The markers shown on single Manhattan plots for any trait are predicted by ISIS EM-BLASSO for which Manhattan plot is not generated.

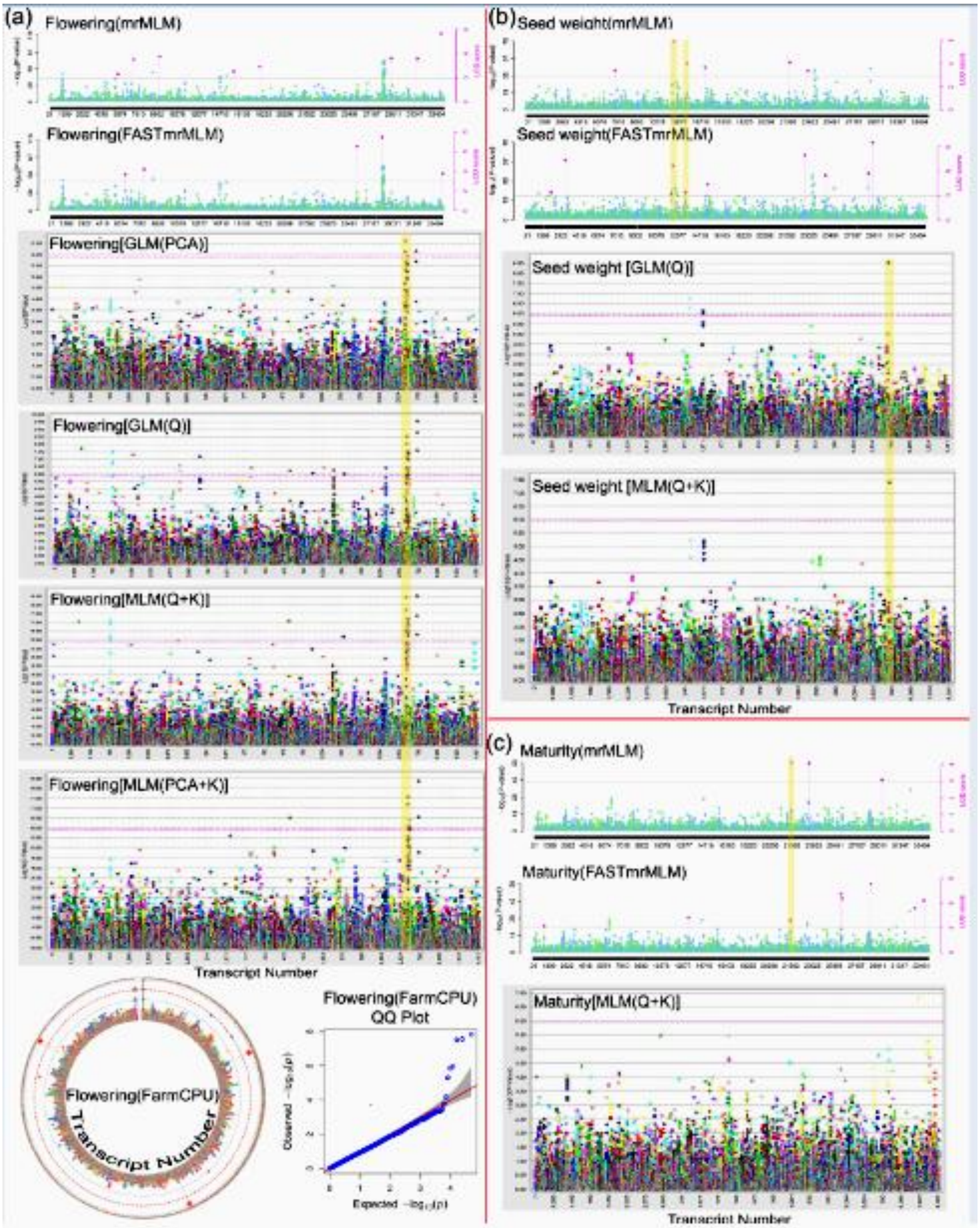

Figure S4. Significantly associated markers for (A) flowering, (B) seed weight and (C) maturity shown on the Manhattan plots, predicted by various models using the phenotypic dataset collected from Delhi in the year 2020. The highlighted regions show the consistent markers predicted by at least two models. The markers shown on single Manhattan plots for any trait are predicted by ISIS EM-BLASSO for which Manhattan plot is not generated.

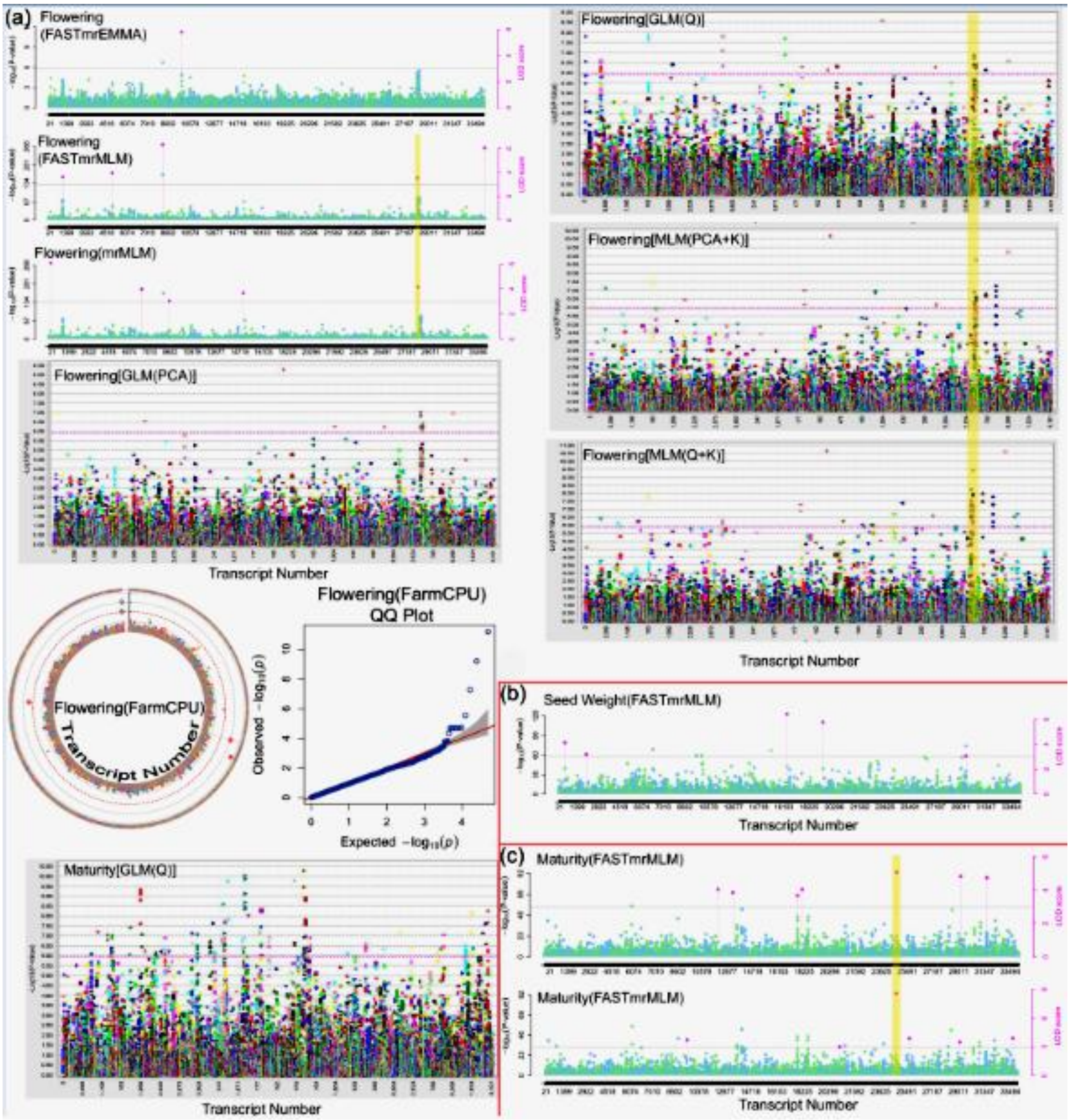

Figure S5. Significantly associated markers for (A) flowering, (B) seed weight and (C) maturity shown on the Manhattan plots, predicted by various models using the phenotypic dataset collected from Delhi in the year 2021. The highlighted regions show the consistent markers predicted by at least two models. The markers shown on single Manhattan plots for any trait are predicted by ISIS EM-BLASSO for which Manhattan plot is not generated.

Supplementary Tables

Table S1. The detailed available passport information on 100 considered accessions including date of collection, site of origin and cultivar name.

Accession	Date of collection	Collector Number	Cultivar Name	Village	Mandal	District	State	Country	Latitude	Longitude
EC16167	07-03-60	NA	Var. yang-liu-tou	NA	NA	NA	NA	China	NA	NA
IC567233	11-14-08	JAS/08-09	NBPGRT/2008-83	Betapur	NA	M.Andaman	Andaman and Nicobar Islands	India	12.717	92.9
IC384034	10-30-02	SSK-166	Thivirlu	Peddalowa/ Kanivada	NA	East Godavari	Andhra Pradesh	India	17.47	81.49
IC426783	12-04-03	BAR-064	Bobberlu	Killoyi	Mandasa	Srikakulam	Andhra Pradesh	India	18.297	83.8968
IC426778	12-04-03	BAR-055-2	Pedda bobberlu	Singapuram	Mandasa	Srikakulam	Andhra Pradesh	India	18.273	84.0048
IC426789	12-06-03	BAR-143-1	Judumulu	Sathinaguda	Seethapeta	Srikakulam	Andhra Pradesh	India	18.273	84.0048
IC343841	12-22-01	SKN-032	NA	Jamthota	NA	Srikakulam	Andhra Pradesh	India	18.721	84.477
IC343929	12-25-01	SKN-120	Minumulu	12th mile	NA	Visakhaptanam	Andhra Pradesh	India	17.687	83.2185
IC350374	12-28-01	RSMP-841	Timmerlu	Chintapalli	NA	Vishakhapatnam	Andhra Pradesh	India	17.687	83.2185
IC353877	01-29-02	SMAR-909	Timerlu	Potaram	NA	Vishakhapatnam	Andhra Pradesh	India	17.687	83.2185
IC369607	02-16-02	NDS-201	Cuttingulu	Mucherlavalasa	NA	Vizianagaram	Andhra Pradesh	India	18.188	83.0546
IC350127	01-19-02	BKSB-248	Pincha	Phanyak	NA	Changlang	Arunachal Pradesh	India	27.742	96.6424
IC433853	03-13-04	DRLT-1732	Moshum	Loiliang	Lohit	Lohit	Arunachal Pradesh	India	27.98	96.5975

IC538983	10-22-05	SKS-126	Amuperung	Pinegrove	NA	Lower Subansiri	Arunachal Pradesh	India	27.1	93.5
IC435833	04-09-04	DRLT-1956	Andeyu	New Aroppo	Upper Dibang	Upper Dibang Valley	Arunachal Pradesh	India	28.733	95.6166
IC19351	03-31-73	NIC NO.-100	NA	Daringibadi	NA	Phulbani	Bihar	India	20.134	84.0167
IC521356	02-11-04	RKH-7	Moth	Balrina(Jandhota)	NA	Bilaspur	Himachal Pradesh	India	31.371	76.6713
IC362094	11-21-99	BDS-2848	Moong	Sahar	NA	Mandi	Himachal Pradesh	India	31.717	76.9166
IC469177	01-01-97	CMX-68P-1	Hara moong	NA	NA	Shimla	Himachal Pradesh	India	31.197	77.6327
IC469185	01-01-97	CXM8-1-1	Hara moong	NA	NA	Shimla	Himachal Pradesh	India	31.197	77.6327
IC326994	10-29-01	SKY-1022	Ganga-Jamuna	Jaunaji	NA	Solan	Himachal Pradesh	India	30.917	77.1166
IC129080	NA	NKG-110(A)	NA	NA	NA	NA	Himachal Pradesh	India		
IC369230	11-15-02	VKG-23/51	Laldhusri	Kamaishela/Bengabad	NA	Giridih	Jharkhand	India	24.3	86.35
IC15642	06-19-72	NA	NA	Netarhat	NA	Latehar	Jharkhand	India	23.756	84.3542
IC15663	06-19-70	NIC No.-09	NA	Lithipara	NA	Pakur	Jharkhand	India	24.634	87.8501
IC15664	06-19-72	NA	NA	Lithipara	NA	Pakur	Jharkhand	India	24.634	87.8501
IC373406	10-27-02	NR/02-141	NA	Sakleshpur	NA	S.Kanara	Karnataka	India	12.844	75.2479
IC273820	04-27-00	IGR-6-2000-6	Bhadi	Lamker	NA	Bastar	Madhya Pradesh	India	19.207	81.9339
IC618587	02-06-16	NA	Jawahar Rice bean 2 (JRBJ 05-4)	JNKVV	NA	Jabalpur	Madhya Pradesh	India	23.182	79.9864
IC26962	01-22-77	NA	NA	None	NA	Tamenglong	Manipur	India	24.764	93.8557
IC352944	12-16-01	BKSB-149	Chakwai achaouba	ICAR (RC), Manipur	NA	Imphal East	Manipur	India	24.817	93.95
IC352853	09-12-01	BKSB-122	Chakwi achouba	Nagarup	NA	Imphal East	Manipur	India	24.817	93.95
IC16799	09-11-72	NIC No.-98	NA	Tonibari	NA	SENAPATI	Manipur	India	25.208	94.0397
IC16796	12-30-72	NA	NA	Tonibari	NA	SENAPATI	Manipur	India	25.208	94.0397

IC352931	12-14-01	BKSB-145	Chakhawai	Kakching	NA	Thoubal	Manipur	India	24.483	93.9833
IC16706	09-11-72	NA	NA	B.Kalgong	NA	Ukhrul	Manipur	India	25.017	94.4311
IC129062	12-31-89	CXM-12P3-1	NA	NA	NA	NA	Manipur	India		
IC350791	07-01-02	BKSB-221	Tohja	Lumkhudung	NA	Jaintia Hill	Meghalay a	India	25.333	92.6666
IC129038	12-31-89	RXS-27-P3	NA	NA	NA	Shillong	Meghalay a	India	25.368	91.7539
IC144701	NA	RXS-4-P-3	NA	NA	NA	Shillong	Meghalay a	India	25.368	91.7539
IC551632	03-15-07	LRB-70	Raj moong	NA	NA	Shillong	Meghalay a	India	25.368	91.7539
IC551711	03-15-07	Vum-85	Raj moong	NA	NA	Shillong	Meghalay a	India	25.368	91.7539
IC144685	NA	CXN-16-P-1-1	NA	NA	NA	Shillong	Meghalay a	India	25.368	91.7539
IC144695	NA	CXN-42-P-2-1	NA	NA	NA	Shillong	Meghalay a	India	25.368	91.7539
IC112381	NA	NA	NA	NA	NA	Shillong	Meghalay a	India	25.368	91.7539
IC112383	NA	NA	NA	NA	NA	Shillong	Meghalay a	India	25.368	91.7539
IC569120	01-11-08	M-006	Rymbai-ja	NA	NA	West Khasi Hills	Meghalay a	India	25.34	91.53
IC141074	NA	BD-139-D	NA	NA	NA	NA	Meghalay a	India	NA	NA
IC129069	NA	CXM-11-P5-1	NA	NA	NA	NA	Meghalay a	India	NA	NA
IC298121	01-03-00	BKSB-70	Bethe	Kaishang	NA	Serchhip	Mizoram	India	23.295	92.9376
IC554711	05-01-06	NRB-21	Naga dal	NA	NA	Khonoma	Nagaland	India	25.652	94.023
IC423374	12-23-03	APRB-9	anukchama	Chuntia	NA	Mokokchung	Nagaland	India	26.333	94.5333
IC129089	NA	D-19	NA	NA	NA	NA	Nagaland	India		
IC569073	08-12-08	HNS/RS-113	Simba	Muniguda	Munigud a	Kalahandi	Odisha	India	19.914	83.1649
IC342229	12-06-00	ORB-49	Dangarrani	Aligaon	NA	Koraput	Odisha	India	18.856	82.7347
IC311934	12-16-00	SS-143	Kutting	Sabarnagiri	NA	Phussari	Odisha	India	20.134	84.0167

IC342242	08-04-00	ORB-66	Dangarrani	Bhamarjod	NA	Rayagada	Odisha	India	19.362	83.5144
IC521177	03-23-05	VUM-22	NA	NA	NA	NA	Others	India	NA	NA
IC521224	03-23-05	BRS-1-A	NA	NA	NA	NA	Others	India	NA	NA
IC521039	03-23-05	LRB-282	NA	NA	NA	NA	Others	India	NA	NA
IC521146	03-23-05	LRB-466	NA	NA	NA	NA	Others	India	NA	NA
IC521156	03-23-05	LRB-510	NA	NA	NA	NA	Others	India	NA	NA
IC140810	NA	RBL-50	NA	NA	NA	NA	Punjab	India	NA	NA
IC137205	NA	RBL-70	NA	NA	NA	NA	Punjab	India	NA	NA
IC557317	12-16-07	BK-07-39	Mashiam dal	Gangtok Market	NA	East Sikkim	Sikkim	India	27.293	88.6781
IC557283	11-12-07	BK-07-05	Mashiam dal	Pachyekhani	NA	East Sikkim	Sikkim	India	27.293	88.6781
IC129097	NA	RXS-58-P3-2	NA	NA	NA	NA	Sikkim	India	NA	NA
IC129078	NA	A-1350A	NA	NA	NA	NA	Sikkim	India	NA	NA
IC621844	11-15-16	AD-16/87	Be-Te	Behliangchhip	Vanghmun, Jampui Hill	North Tripura	Tripura	India	23.93	92.27
IC316123	11-12-00	VRS-OM-1912	Muad	Dewaraniya	NA	Bareilly	Uttar Pradesh	India	28.453	79.4168
IC129115	NA	VT-72/844-A	NA	NA	NA	NA	Uttar Pradesh	India		
IC621805	09-27-16	KCB/RSR/PKM-13	Dhuans	Bugoti/Chakarata	NA	Dehradun	Uttarakhand	India	30.317	78.0322
IC116113	03-31-89	ARB-83(114-D)	NA	Bhowali	NA	Nainital	Uttarakhand	India	29.38	79.4636
IC116118	03-31-89	CXN-7-B	NA	Bhowali	NA	Nainital	Uttarakhand	India	29.38	79.4636
IC137169	10-10-85	PRR-8801-A	Ghurush	Ranichauri	NA	Tehri	Uttarakhand	India	30.301	78.5661
IC351534	12-15-01	BKSB-166	Thulibhatamas	Damri Tari	NA	Darjeeling	West Bengal	India	27.041	88.2663
IC552979	01-29-04	KRB-96	Gaimung	Garubathan	NA	Darjeeling	West Bengal	India	27.041	88.2663
IC2074	04-18-53	NIC No.-100	NA	Kalimpong	NA	Darjeeling	West Bengal	India	27.041	88.2663
IC351508	12-13-01	BKSB-160	Gala dal	Rangsang	NA	Darjeeling	West Bengal	India	27.041	88.2663

IC552995	06-02-04	KRB-113	Gaimung	Banarhat	NA	Jalpaiguri	West Bengal	India	26.544	88.7205
IC552977	01-29-04	KRB-94	Gaimung	Garubathan	NA	Jalpaiguri	West Bengal	India	26.544	88.7205
IC552970	01-25-04	KRB-87	Gaimung	Madarihat	NA	Jalpaiguri	West Bengal	India	26.544	88.7205
IC449234	05-12-04	KG/PB-10	Maskalai	Brajalaltola	Manikchak	Maldah	West Bengal	India	25.179	88.2461
IC564858	02-08-05	KRB-153	Gaimung	Berhampur	NA	Murshidabad	West Bengal	India	24.161	88.2328
IC564865	02-10-05	KRB-161	Gaimung	Raningar-I	NA	Murshidabad	West Bengal	India	24.161	88.2328
IC545607	05-01-03	KRB-64	Gaimung	Balarampur	NA	Purulia	West Bengal	India	23.183	86.4833
IC2567	NA	NA	NA	NA	NA	NA	NA	India	NA	NA
EC114075	07-08-75	NA	((1085)w5a)	NA	NA	NA	NA	Indonesia	NA	NA
EC934343	02-11-17	JP No223033	NA	NA	NA	NA	NA	Japan	NA	NA
EC934368	02-11-17	JP No251211	NA	NA	NA	NA	NA	Japan	NA	NA
EC934417	02-11-17	JP No31442	NA	NA	NA	NA	NA	Japan	NA	NA
EC18136	04-18-61	Pokhra village	Moshang	NA	NA	NA	NA	Nepal	NA	NA
EC18183	04-18-61	Bhumri	Shilli	NA	NA	NA	NA	Nepal	NA	NA
EC18261	04-18-61	Matikhana	NA	NA	NA	NA	NA	Nepal	NA	NA
EC615198	03-13-08	NA	Hong kong HK1	NA	NA	NA	NA	Taiwan	NA	NA
EC615199	03-13-08	NA	Hong Kong HK2	NA	NA	NA	NA	Taiwan	NA	NA
EC615195	03-13-08	NA	Mogimass	NA	NA	NA	NA	Taiwan	NA	NA
EC615201	03-13-08	NA	NA	NA	NA	NA	NA	Taiwan	NA	NA
EC18222	NA	Dumbri Khola	NA	NA	NA	NA	NA	India	NA	NA

EC1843	NA	NA	NA	NA	NA	NA	NA	NA	NA	NA

Table S2. The number of bases and reads before and after filtration from the transcriptome data of 100 accessions of rice bean

**Sample**	**Total reads**	**Total bases**	**Reads after filtration**	**Bases after filtration**
IC362094	36819636	5559765036	33717652	4917640072
IC129062	31444232	4748079032	28828384	4209254643
IC112381	22706738	3428717438	20509398	2955955654
IC384034	23352124	3526170724	21259702	3087996122
IC2567	21459328	3240358528	20279740	3054448536
EC16167	30497154	4605070254	29401510	4416367942
IC144701	27301512	4122528312	26134908	3928259800
IC15663	126603058	19117061758	115922528	16917765535
IC373406	51919498	7839844198	47263438	6857884444
IC449234	45084298	6807728998	41335656	6031928670
EC18261	43577642	6580223942	41687614	6291179824
IC129038	39809460	6011228460	38096944	5749476994
IC469177	38935994	5879335094	35225834	5097728408
IC521224	37776142	5704197442	36373266	5457704369
IC140810	37679322	5689577622	36325710	5472247386
IC521356	37021004	5590171604	33918838	4935017567
IC426783	36688940	5540029940	35425498	5334800800
EC18183	36395406	5495706306	33170848	4796940704
EC114075	36006220	5436939220	32464060	4705669121
IC521146	35784686	5403487586	31214402	4664653632
IC552977	35626502	5379601802	34353016	5129300225

IC129078	35539446	5366456346	33704394	5073741377
IC353877	35157496	5308781896	31699606	4657735376
IC521156	35134458	5305303158	33693888	5079088773
IC116118	35037812	5290709612	32025558	4685313810
IC618587	34896772	5269412572	31644576	4587161587
EC1843	34736162	5245160462	33508626	5043663700
EC934417	34554966	5217799866	31462564	4600598780
IC521039	34518194	5212247294	33325856	5020557003
IC426778	34431552	5199164352	33222606	4983874750
IC316123	34257030	5172811530	32855888	4958859535
IC326994	34212624	5166106224	32394088	4877575843
IC369230	34173840	5160249840	31187874	4566892334
IC2074	34087934	5147278034	31190348	4546121372
IC129069	34009918	5135497618	32521238	4908714330
IC141074	33347404	5035458004	31732666	4779839884
IC564858	33174806	5009395706	30512488	4466591862
EC615199	33164412	5007826212	30273646	4386464052
IC352944	33076738	4994587438	29885212	4349451915
IC351534	32991796	4981761196	28982862	4167666586
EC615195	32988670	4981289170	29991590	4352980144
IC426789	32947076	4975008476	31460036	4737617377
IC552995	32718744	4940530344	29647226	4283996199
IC350791	32636006	4928036906	29290636	4257385288
IC15664	32322948	4880765148	31078232	4680264266
IC350374	32279416	4874191816	29334150	4207053939
IC569120	32177150	4858749650	30998222	4668830545
IC369607	31836728	4807345928	30611834	4604553867
IC621844	31642204	4777972804	28977744	4178109392
IC144685	31496144	4755917744	30074108	4527290004
EC18136	31445624	4748289224	28566304	4127606621

IC342229	31249634	4718694734	28487838	4153712458
EC934343	31188932	4709528732	28458388	4135230880
IC569073	31096630	4695591130	28407118	4130850338
IC551711	30703792	4636272592	27501270	3973834404
IC129080	30463206	4599944106	29410944	4420157805
IC16706	30222338	4563573038	29185454	4399891346
IC521177	30101188	4545279388	28629864	4321614374
IC144695	30049828	4537524028	28840420	4343279332
IC351508	29875244	4511161844	27207196	3952148272
IC552970	29243756	4415807156	28025266	4222443288
IC137169	29138546	4399920446	26818154	3902165556
IC567233	28763476	4343284876	26442498	3841696091
EC615198	28745474	4340566574	26198000	3812270726
IC554711	28721980	4337018980	27393870	4122834124
IC352931	28648924	4325987524	27484120	4148391732
IC621805	28310500	4274885500	27181650	4088459405
EC18222	28088900	4241423900	26545144	4005428780
IC112383	28036112	4233452912	26987132	4047570091
IC26962	28004548	4228686748	26665484	4016366029
IC343929	27983170	4225458670	26691796	4014354268
IC552979	27803498	4198328198	26753842	4009222381
IC15642	27790330	4196339830	24455418	3480527279
EC934368	27786944	4195828544	25022368	3629493271
IC435833	27576654	4164074754	24718710	3540437024
IC116113	27532386	4157390286	26500020	3978709315
IC557283	27441632	4143686432	24908386	3597109502
IC564865	27356544	4130838144	26212870	3955944556
IC538983	27296446	4121763346	24840326	3590888833
IC350127	27258134	4115978234	26251276	3934637119
IC16799	27210738	4108821438	23847258	3424257212

IC298121	27182044	4104488644	26111506	3918039404
IC16796	27178530	4103958030	25844260	3892204051
IC19351	27149416	4099561816	25720204	3879244220
IC129089	26956328	4070405528	25589760	3851215287
IC433853	26680642	4028776942	25743082	3866843031
IC557317	26647174	4023723274	25365606	3827494394
IC545607	26160306	3950206206	23787988	3425619160
IC137205	25979038	3922834738	23979038	3405890365
IC342242	25498482	3850270782	23141442	3361704154
IC469185	25189682	3803641982	22827010	3300204216
IC311934	24607454	3715725554	22341342	3212799348
IC273820	24381096	3681545496	22180804	3205790264
IC129115	24212892	3656146692	21751190	3143688357
EC615201	24206134	3655126234	21377814	3057066224
IC343841	23783392	3591292192	21756340	3158586198
IC129097	23186710	3501193210	21009328	3036510510
IC423374	22808980	3444155980	20631752	2962221575
IC352853	22077506	3333703406	20025582	2908927223
IC551632	21141650	3192389150	19321214	2811402893

Table S3. Associated Markers for flowering, maturity and seed weight predicted using phenotypic data of Almora location in the year 2021.

Marker ID[a]	SNP[#]	Transcript ID	Position	Models[b]	Trait(s)	LOD[#]	MAF[#]	R^2
SVUTC21295_283	G/A	VUTC21295	283	6	Flowering, Maturity	*LOD:* 3.69 to 7.50	0.110	Flowering: 6.35 Maturity: 20.45
SVUTC22319_77	T/C	VUTC22319	77	6, 8	Flowering	*LOD:*4.58 to 5.74	0.284	5.92 to 7.31
SVUTC31327_3358	T/G	VUTC31327	3358	6, 8	Flowering	*LOD:*5.32 to 6.18	0.165	23.22 to 24.54
SVUTC05378_2089	T/C	VUTC05378	2089	6, 8	Maturity	*LOD:*3.89 to 8.60	0.077	9.73 to 7.15
SVUTC21831_6083	G/A	VUTC21831	6083	5,6	Maturity	*LOD:*5.49 to 16.21	0.060	15.12 to 19.28
SVUTC01132_259	A/G	VUTC01132	259	6, 7	Seed weight	*LOD:* 3.09 to 5.23	0.396	7.16 to 7.95
SVUTC06850_2182	C/A	VUTC06850	2182	6, 8	Seed weight	*LOD:*3.06 to 6.23	0.192	5.32 to 7.15
SVUTC09951_1960	C/A	VUTC09951	1960	6,8	Seed weight	*LOD:*3.97 to 4.78	0.3032	6.79 to 8.69
SVUTC23058_1028	A/T	VUTC23058	1028	6,8	Seed weight	*LOD:*6.26 to 13.15	0.140	17.22 to 21.87
SVUTC24042_2561	T/A	VUTC24042	2561	6,8	Seed weight	*LOD:*4.13 to 5.71	0.318	1.66 to 2.29
SVUTC24699_1593	G/T	VUTC24699	1593	6,8	Seed weight	*LOD:*3.16 to 5.69	0.081	3.94 to 5.55

[a] The marker names in bold italics are associated with more than one trait.

[b] 1. GLM (Q), 2. GLM (PCA)], 3. MLM (Q+K), 4. MLM (PCA+K), 5. mrMLM, 6. FASTmrMLM, 7. FASTmrEMMA, 8. ISIS EMBLASSO, 9. FarmCPU, 10. BLINK.

[#] *SNP:* single nucleotide polymorphism, LOD: logarithm of the odds, MAF: minor allele frequency

Table S4. Associated Markers for flowering, maturity and seed weight predicted using phenotypic data of Delhi location in the year 2020.

Marker ID	SNP #	Transcript ID	Position	Models[a]	Trait(s)	-log10(P)/ LOD#	MAF#	R^2
SVUTC02166_197	G/A	VUTC02166	197	1,3	Flowering	*-log$_{10}$(P):*7.01 to 7.7	0.071	33 to 35
SVUTC04837_563	C/T	VUTC04837	563	1,3	Flowering	*-log10(P):*7.10 to 7.51	0.286	29 to 31
SVUTC04837_620	T/C	VUTC04837	620	1,3	Flowering	*-log10(P):*6.90 to 7.47	0.296	28 to 30
SVUTC04837_772	G/T	VUTC04837	772	1,3	Flowering	*-log10(P):*6.12 to 6.68	0.109	26 to 28
SVUTC04837_782	A/C	VUTC04837	782	1,3	Flowering	*-log10(P):*6.14 to 6.78	0.096	27 to 29
SVUTC04837_786	G/C	VUTC04837	786	1,3	Flowering	*-log10(P):*6.14 to 6.78	0.074	27 to 29
SVUTC06321_645	C/T	VUTC06321	645	1,6,9,10,	Flowering	*-log10(P):*4.40 to 8.44 *LOD:*3.67	0.110	7.07 to 29
SVUTC14443_583	T/C	VUTC14443	583	9,10	Flowering	*-log10(P):*7.56 to 8.3	0.095	NA*
SVUTC15693_1885	T/C	VUTC15693	1885	5,8	Flowering	*-log10(P):*4.51 to 4.51 *LOD:*3.7705 to 3.7725	0.220	8.22 to 16.28
SVUTC18323_834	G/A	VUTC18323	834	1,3,4	Flowering	*-log10(P):*6.21 to 7.06	0.067	28 to 34
SVUTC22119_491	C/A	VUTC22119	491	1,3	Flowering	*-log10(P):*5.99 to 6.15	0.061	27 to 28
SVUTC26171_1046	A/C	VUTC26171	1046	6,8	Flowering	*LOD:*3.67 to 7.65	0.133	13.99 to 19.49
SVUTC28154_1391	G/T	VUTC28154	1391	1,2, 3,4	Flowering	*-log10(P):*6.12 to 8.00	0.076	21 to 36
SVUTC28154_1530	A/G	VUTC28154	1530	1,3	Flowering	*-log10(P):*6.45 to 7.05	0.071	29 to 31
SVUTC28154_1646	G/A	VUTC28154	1646	6,8,9,10 1,2, 3,4	Flowering	*-log10(P):*6..60 to 8.51 LOD: 7.49 to 8.58	0.065	19 to 35
SVUTC28154_4577	A/C	VUTC28154	4577	1,3	Flowering	*-log10(P):*6.38 to 6.50	0.055	26 to 27
SVUTC28154_4595	T/C	VUTC28154	4595	1,3	Flowering	*-log10(P):*6.38 to 6.50	0.055	26 to 27
SVUTC28154_4614	A/C	VUTC28154	4614	1,3	Flowering	*-log10(P):*6.38 to 6.50	0.055	26 to 27
SVUTC28154_4619	T/A	VUTC28154	4619	1,3	Flowering	*-log10(P):*6.38 to 6.50	0.055	26 to 27
SVUTC28154_4769	G/A	VUTC28154	4769	1,3	Flowering	*-log10(P):*6.38 to 6.50	0.055	26 to 27
SVUTC28155_2560	G/T	VUTC28155	2560	1,3	Flowering	*-log10(P):*6.2 to 6.75	0.063	27 to 29
SVUTC28163_1003	T/C	VUTC28163	1003	1,3	Flowering	*-log10(P):*6.38 to 6.50	0.055	26 to 27

SVUTC28164_1018	G/A	VUTC28164	1018	1,3	Flowering	*-log10(P):*6.38 to 6.50	0.055	26 to 27
SVUTC28164_684	C/T	VUTC28164	684	1,3	Flowering	*-log10(P):*6.38 to 6.50	0.055	26 to 27
SVUTC28164_974	G/T	VUTC28164	974	1,3	Flowering	*-log10(P):*6.38 to 6.50	0.055	26 to 27
SVUTC28165_2013	C/T	VUTC28165	2013	1,3	Flowering	*-log10(P):*6.24 to 6.74	0.063	27 to 29
SVUTC28165_2563	G/A	VUTC28165	2563	1,3	Flowering	*-log10(P):*6.36 to 6.44	0.056	27 to 27
SVUTC28165_2595	G/A	VUTC28165	2595	1,3	Flowering	*-log10(P):*6.44 to 6.51	0.056	27 to 27
SVUTC28165_3265	A/T	VUTC28165	3265	1,3,4	Flowering	*-log10(P):*6.10 to 7.55	0.060	22 to 30
SVUTC28181_1427	A/G	VUTC28181	1427	1,3	Flowering	*-log10(P):*6.38 to 6.50	0.055	26 to 27
SVUTC28181_1461	T/C	VUTC28181	1461	1,3	Flowering	*-log10(P):*6.36 to 6.44	0.056	27 to 27
SVUTC28183_2077	G/A	VUTC28183	2077	1,3,4	Flowering	*-log10(P):*6.10 to 7.55	0.060	22 to 30
SVUTC28185_6557	T/C	VUTC28185	6557	1,3,4	Flowering	*-log10(P):*6.40 to 7.30	0.058	24 to 30
SVUTC28186_2590	C/A	VUTC28186	2590	1,3	Flowering	*-log10(P):*6.38 to 6.50	0.055	26 to 27
SVUTC28192_1776	G/A	VUTC28192	1776	1,3	Flowering	*-log10(P):*6.10 to 6.35	0.057	26 to 27
SVUTC28192_1815	T/G	VUTC28192	1815	1,3	Flowering	*-log10(P):*7.03 to 7.24	0.063	20 to 30
SVUTC28192_1930	A/G	VUTC28192	1930	1,3	Flowering	*-log10(P):*6.38 to 6.50	0.055	26 to 27
SVUTC28192_2307	G/A	VUTC28192	2307	1,3,4	Flowering	*-log10(P):*7.10 to 7.55	0.060	22 to 30
SVUTC28196_1051	T/A	VUTC28196	1051	1,3	Flowering	*-log10(P):*6.42 to 6.49	0.057	27 to 27
SVUTC28201_1062	G/A	VUTC28201	1062	1,3	Flowering	*-log10(P):*7.44 to 787	0.065	28 to 30
SVUTC28201_425	A/G	VUTC28201	425	1,3	Flowering	*-log10(P):*6.81 to 7.35	0.066	28 to 30
SVUTC28201_516	C/A	VUTC28201	516	1,3	Flowering	*-log10(P):*6.80 to 7.35	0.066	28 to 30
SVUTC28201_680	A/T	VUTC28201	680	1,3	Flowering	*-log10(P):*6.87 to 7.44	0.065	28 to 30
SVUTC28201_76	T/C	VUTC28201	76	1,3	Flowering	*-log10(P):*6.23 to 6.96	0.066	28 to 31
SVUTC28966_1949	C/A	VUTC28966	1949	1,3, 7	Flowering	*-log10(P):*7.38 to 8.51 *LOD:*3.06-9.52	0.250	6.81 to 34
SVUTC29107_1520	A/C	VUTC29107	1520	1,3,4	Flowering	*-log10(P):*6.54 to 8.78	0.056	26 to 37
SVUTC29109_1026	G/T	VUTC29109	1026	1, 2, 3,4	Flowering	*-log10(P):*6.16 to 9.55	0.059	20 to 39
SVUTC29109_858	C/G	VUTC29109	858	1,3	Flowering	*-log10(P):*6.13 to 7.59	0.065	28 to 33
SVUTC34794_4308	G/A	VUTC34794	4308	6,8	Flowering	*-log10(P):*4.50 to 4.82/"-lod": 3.76-4.07	0.184	7.58 to 10.91
SVUTC21642_866	T/A	VUTC21642	866	6,5	Maturity	*-log10(P):*4.66 to 4.86	0.142	8.85 to 38.77

						*LOD:*3.9-4.11		
SVUTC26129_1121	T/C	VUTC26129	1121	6,8	Maturity	*-log10(P):*6.18 to 8.01 *LOD:*5.37 to 7.14	0.068	17.16 to 22.68
SVUTC32582_1223	A/G	VUTC32582	1223	1,3	Maturity	*-log10(P):*6.79 to 9.71	0.063	25 to 33
SVUTC33036_2172	G/T	VUTC33036	2172	1,6	Maturity	*-log10(P):* 7.01 *LOD:* 5.39	0.058	9.05 to 32
SVUTC02299_468	G/C	VUTC02299	468	6,7	Seed weight	*LOD:*3.38-3.44	0.293	2.53 to 4.58
SVUTC12393_763	C/T	VUTC12393	763	1,7,5	Seed weight	*-log10(P):*4.40 to 7.54 *LOD:* 3.66 to 6.68	0.274	8.30 to 29
SVUTC13707_7465	C/G	VUTC13707	7465	1, 5	Seed weight	*-log10(P):*4.76 to 6.09 *LOD:* 4.01	0.109	20.42 to 27
SVUTC13707_7487	A/G	VUTC13707	7487	1, 6	Seed weight	*-log10(P):*4.08 to 6.11 *LOD:* 3.37	0.114	6.43 to 27
SVUTC25355_209	A/G	VUTC25355	209	6,8	Seed weight	*-log10(P):*6.30 to 8.77 *LOD:*5.48-7.88	0.219	12.14 to 24.79

[a] 1. GLM (Q), 2. GLM (PCA)], 3. MLM (Q+K), 4. MLM (PCA+K), 5. mrMLM, 6. FASTmrMLM, 7. FASTmrEMMA, 8. ISIS EMBLASSO, 9. FarmCPU, 10. BLINK.[*] As FarmCPU and BLINK don't give R^2 value, NA is written for the markers predicted only by these two methods

[#] *SNP:* single nucleotide polymorphism, LOD: logarithm of the odds, MAF: minor allele frequency

Table S5. Associated Markers for flowering, maturity and seed weight predicted using phenotypic data of Delhi location in the year 2021.

Marker ID[a]	SNP[#]	Transcript ID	Position	Models[b]	Trait(s)	-log10(P0/ LOD[#]	MAF[#]	R^2
SVUTC00230_1049	A/T	VUTC00230	1049	2,4,5	Flowering	*-log10(p):* 6.02 to 7.01<br>*LOD:* 6.063	0.113	16.86 to 22
SVUTC01074_2125	A/G	VUTC01074	2125	3,4	Flowering	*-log10(p):* 6.37 to 7.09	0.118	27
SVUTC01110_689	T/C	VUTC01110	689	1,8	Flowering	*-log10(P):*5.90 to 6.31<br>*LOD:* 5.10	0.070	12.07 to 26
SVUTC04786_495	T/A	VUTC04786	495	3,4	Flowering	*-log10(P):*7.37 to 7.70	0.060	30
SVUTC04786_499	A/T	VUTC04786	499	3,4	Flowering	*-log10(P):*7.18 to 7.52	0.060	29
SVUTC06771_4276	G/C	VUTC06771	4276	2,4	Flowering	*-log10(P):*6.43 to 6.55	0.153	20 to 25
SVUTC08154_978	G/A	VUTC08154	978	6,8	Flowering	*-log10(P):*7.18 to 7.19<br>*LOD:* 6.341 to 6.3495	0.051	29.21 to 33.54
SVUTC09869_1881	C/G	VUTC09869	1881	1,3	Flowering	*-log10(P):*6.28 to 7.81	0.098	35
SVUTC09870_149	C/T	VUTC09870	149	1,7,8,9	Flowering	*-log10(P):*6.95 to 9.18<br>*LOD:* 5.77 to 7.12	0.092	15.04 to 33
SVUTC09871_149	C/T	VUTC09871	149	1,3	Flowering	*-log10(P):*6.11 to 6.33	0.077	28
SVUTC15414_2770	T/C	VUTC15414	2770	1,3,4	Flowering	*-log10(P):*6.30 to 7.29	0.075	25 to 26
SVUTC15414_2854	A/T	VUTC15414	2854	3,4	Flowering	*-log10(P):*6.16 to 6.86	0.081	23
SVUTC15418_711	C/T	VUTC15418	711	1, 3,4	Flowering	*-log10(P):*6.30 to 7.29	0.075	25 to 26
SVUTC17024_838	C/T	VUTC17024	838	1,2,3,4	Flowering	*-log10(P):*6.14 to 10.64	0.111	24 to 34
SVUTC20322_1284	A/G	VUTC20322	1284	3,4	Flowering	*-log10(P):*5.99 to 6.82	0.148	28
SVUTC20322_1298	A/T	VUTC20322	1298	3,4	Flowering	*-log10(P):*5.92 to 5.99	0.160	28
SVUTC20819_606	T/C	VUTC20819	606	1,2	Flowering	*-log10(P):*6.16 to 6.86	0.079	20 to 37
SVUTC25024_1324	A/G	VUTC25024	1324	1,2,3,4	Flowering	*-log10(P):*6.23 to 6.59	0.090	19 to 27
SVUTC25312_4994	A/G	VUTC25312	4994	8,9	Flowering	*-log10(P):*5.66 to 7.27<br>*LOD:* 4.87	0.376	7.76
SVUTC28152_1002	C/T	VUTC28152	1002	1,3	Flowering	*-log10(P):*6.40 to 6.49	0.065	26
SVUTC28154_1391	G/T	VUTC28154	1391	1,2,3,4,6	Flowering	*-log10(P):*4.25 to 822<br>*LOD:* 3.52	0.076	8.74 to 32
SVUTC28154_1530	A/G	VUTC28154	1530	1,2,3,4	Flowering	*-log10(P):*6.17 to 7.53	0.071	20 to 28

SVUTC28154_1646	G/A	VUTC28154	1646	1,2,3,4	Flowering	*-log10(P):*6.18 to 7.19	0.065	20 to 29
SVUTC28165_3265	A/T	VUTC28165	3265	1,2,3,4	Flowering	*-log10(P):*6.00 to 7.16	0.060	18 to 26
SVUTC28183_1588	T/G	VUTC28183	1588	1,2,3,4	Flowering	*-log10(P):*6.00 to 9.27	0.074	19 to 32
SVUTC28183_2077	G/A	VUTC28183	2077	1,2,3,4	Flowering	*-log10(P):*6.00 to 7.16	0.060	18 to 22
SVUTC28185_6557	T/C	VUTC28185	6557	1,2	Flowering	*-log10(P):*5.99 to 6.38	0.058	18 to 25
SVUTC28186_2802	G/C	VUTC28186	2802	3,4	Flowering	*-log10(P):*6.18 to 6.26	0.071	26
SVUTC28192_1815	T/G	VUTC28192	1815	1, 2,3	Flowering	*-log10(P):*6.04 to 7.05	0.063	18 to 27
SVUTC28192_2307	G/A	VUTC28192	2307	1,2,3,4	Flowering	*-log10(P):*6.00 to 7.16	0.060	18 to 26
SVUTC28201_1062	G/A	VUTC28201	1062	1,2,3,4	Flowering	*-log10(P):*6.25 to 7.95	0.065	18 to 26
SVUTC28201_425	A/G	VUTC28201	425	1,2,3,4	Flowering	*-log10(P):*6.26 to 7.90	0.066	18 to 26
SVUTC28201_516	C/A	VUTC28201	516	1,2,3,4	Flowering	*-log10(P):*6.17 to 7.85	0.066	18 to 26
SVUTC28201_680	A/T	VUTC28201	680	1,2,3,4	Flowering	*-log10(P):*6.25 to 7.95	0.065	18 to 26
SVUTC28201_76	T/C	VUTC28201	76	1,2,3,4	Flowering	*-log10(P):*6.17 to 7.82	0.066	19 to 28
SVUTC29109_1026	G/T	VUTC29109	1026	3,4	Flowering	*-log10(P):*6.68 to 7.99	0.059	25
SVUTC29109_858	C/G	VUTC29109	858	3,4	Flowering	*-log10(P):*6.78 to 7.50	0.065	26
SVUTC29946_2497	T/G	VUTC29946	2497	3,4	Flowering	*-log10(P):*6.10 to 6.68	0.087	25
SVUTC29946_2507	A/G	VUTC29946	2507	3,4	Flowering	*-log10(P):*6.64 to 7.31	0.091	28
SVUTC29946_2525	G/C	VUTC29946	2525	3,4	Flowering	*-log10(P):*7.25 to 7.25	0.090	29
SVUTC29946_2663	G/A	VUTC29946	2663	3,4	Flowering	*-log10(P):*6.96 to 7.76	0.077	26
SVUTC31155_1020	A/G	VUTC31155	1020	1,2,3,4	Flowering	*-log10(P):*6.23 to 10.59	0.061	24 to 38
SVUTC12308_931	T/A	VUTC12308	931	1,6	Maturity	*-log10(P):*4.79 to 8.57<br>*LOD:* 4.04	0.163	9.05 to 35
SVUTC18444_3244	C/A	VUTC18444	3244	1,6	Maturity	*-log10(P):*4.7 to 9.45<br>*LOD:* 4.032	0.148	13.41 to 39
SVUTC29349_1348	G/A	VUTC29349	1348	5,6	Maturity	*-log10(P):*4.22 to 5.60<br>*LOD:* 3.49-4.81	0.478	4.65 to 5.24
SVUTC25248_8323	G/A	VUTC25248	8323	5,6,8	Maturity,<br>Seed weight	*-log10(P):*5.22 to 9.50<br>*LOD:* 4.44-8.59	0.159	6.37 to 31.87
SVUTC00580_1033	T/C	VUTC00580	1033	6,8	Seed weight	*-log10(P):*3.75 to 4.88	0.085	2.15 to 5.59

SVUTC16440_582	A/G	VUTC16440	582	6,8	Seed weight	*LOD:* 3.05 to 4.12<br>*-log10(P):*4.59 to 7.25<br>*LOD:* 3.84 to 7.25	0.074	5.88 to 22.38
SVUTC19671_1032	G/A	VUTC19671	1032	6,8	Seed weight	*-log10(P):*4.48 to 6.57<br>*LOD:* 3.74 to 5.75	0.087	6.99 to 18.92

[a] The marker names in bold italics are associated with more than one trait.

[b] 1. GLM (Q), 2. GLM (PCA)], 3. MLM (Q+K), 4. MLM (PCA+K), 5. mrMLM, 6. FASTmrMLM, 7. FASTmrEMMA, 8. ISIS EMBLASSO, 9. FarmCPU, 10. BLINK.

[#] *SNP:* single nucleotide polymorphism, LOD: logarithm of the odds, MAF: minor allele frequency

Table S6. The chromosomal localization of markers with respect to the genome of the rice bean cultivar FF25

Transcript (Marker position with left and right flanking coordinates)	Subject (FF25) accession	Query Start	Query end	Subject start	Subject end	e-value	Bit score	Percent Identity	Query coverage	Chromosome number (FF25)	Chromosomal position
VuTC00230_1049_999_1099	CM045811.1	1	101	9631952	9631852	1.19E-46	187	100	100	3	9631902
VuTC00580_1033_983_1083	CM045811.1	1	101	4772554	4772454	1.19E-46	187	100	100	3	4772504
VuTC01074_2125_2075_2175	CM045811.1	1	101	1173835	1173935	1.19E-46	187	100	100	3	1173885
VuTC01110_689_639_739	CM045811.1	1	71	769885	769815	5.65E-30	132	100	100	3	769835
VuTC01132_259_209_309	CM045811.1	1	101	695239	695339	1.19E-46	187	100	100	3	695289
VuTC02283_1297_1247_1347	CM045811.1	1	101	30937366	30937466	1.19E-46	187	100	100	3	30937416
VuTC02299_468_418_518	CM045811.1	1	101	30795162	30795062	1.2E-41	171	97.03	100	3	30795112
VuTC02300_1585_1535_1635	CM045811.1	1	87	30758380	30758294	3.35E-37	156	98.851	86	3	30758330
VuTC04786_495_445_545	CM045809.1	24	101	28992314	28992237	7.26E-34	145	100	77	1	28992287
VuTC04786_499_449_549	CM045809.1	20	101	28992314	28992233	4.34E-36	152	100	81	1	28992283
VuTC04837_563_513_613	CM045809.1	1	101	30497299	30497399	1.19E-46	187	100	100	1	30497349
VuTC04837_620_570_670	CM045809.1	1	101	30497356	30497456	1.19E-46	187	100	100	1	30497406
VuTC04837_772_722_822	CM045809.1	1	101	30497508	30497608	1.19E-46	187	100	100	1	30497558
VuTC04837_782_732_832	CM045809.1	1	101	30497518	30497618	5.53E-45	182	99.01	100	1	30497568
VuTC04837_786_736_836	CM045809.1	1	101	30497522	30497622	5.53E-45	182	99.01	100	1	30497572
VuTC05378_2089_2039_2139	CM045815.1	1	101	4657299	4657199	5.53E-45	182	99.01	100	7	4657249
VuTC06321_645_595_695	CM045817.1	1	101	6378333	6378433	1.19E-46	187	100	100	9	6378383
VuTC06771_4276_4226_4326	CM045814.1	1	101	26027670	26027570	1.19E-46	187	100	100	6	26027620
VuTC06850_2182_2132_2232	CM045809.1	1	101	48733273	48733373	1.19E-46	187	100	100	1	48733323
VuTC06910_1648_1598_1698	CM045818.1	1	74	9729407	9729480	2.63E-28	126	97.297	73	10	9729457
VuTC06910_1702_1652_1752	CM045818.1	24	101	9729475	9729552	1.57E-30	134	97.436	77	10	9729502
VuTC06910_1746_1696_1796	CM045818.1	1	101	9729496	9729596	2.57E-43	176	98.02	100	10	9729546
VuTC06910_177_127_227	CM045818.1	1	101	9727974	9728074	5.53E-45	182	99.01	100	10	9728024
VuTC07229_692_642_742	CM045812.1	17	101	5850084	5850168	9.32E-38	158	100	84	4	5850118
VuTC08788_874_824_924	CM045814.1	1	62	29005354	29005293	2.65E-23	110	98.387	100	6	29005304
VuTC09869_1881_1831_1931	CM045814.1	1	101	37159630	37159530	1.19E-46	187	100	100	6	37159580
VuTC09870_149_99_199	CM045814.1	1	101	37164076	37163976	1.19E-46	187	100	100	6	37164026
VuTC09951_1960_1910_2010	CM045814.1	1	101	37648884	37648984	2.57E-43	176	98.02	100	6	37648934
VuTC12308_931_881_981	CM045810.1	1	101	42038354	42038254	1.19E-46	187	100	100	2	42038304

VuTC12393_763_713_813	CM045810.1	46	101	40905743	40905688	1.23E-21	104	100	100	2	40905738
VuTC12822_2877_2827_2927	CM045810.1	1	101	34427114	34427214	1.19E-46	187	100	100	2	34427164
VuTC13707_7465_7415_7515	CM045818.1	1	101	2381645	2381745	1.19E-46	187	100	100	10	2381695
VuTC13707_7487_7437_7537	CM045818.1	1	101	2381667	2381767	1.19E-46	187	100	100	10	2381717
VuTC14443_583_533_633	CM045810.1	1	101	4826509	4826609	1.19E-46	187	100	100	2	4826559
VuTC15414_2770_2720_2820	CM045815.1	1	101	19788538	19788638	1.19E-46	187	100	100	7	19788588
VuTC15414_2854_2804_2896	CM045815.1	1	93	19788622	19788714	2.98E-42	172	100	100	7	19788672
VuTC15418_711_661_761	CM045815.1	1	101	19953618	19953518	1.19E-46	187	100	100	7	19953568
VuTC15693_1885_1835_1935	CM045817.1	1	101	3273097	3272997	1.19E-46	187	100	100	9	3273047
VuTC16440_582_532_632	CM045816.1	1	101	39541747	39541847	1.19E-46	187	100	100	8	39541797
VuTC17024_838_788_888	CM045816.1	1	101	22745909	22746009	1.19E-46	187	100	100	8	22745959
VuTC18323_834_784_884	CM045816.1	1	101	8513761	8513661	1.19E-46	187	100	100	8	8513711
VuTC18444_3244_3194_3294	CM045816.1	1	101	7456468	7456568	2.57E-43	176	98.02	100	8	7456518
VuTC19671_1032_982_1082	CM045813.1	1	101	33927832	33927932	1.19E-46	187	100	100	5	33927882
VuTC20322_1284_1234_1334	CM045819.1	1	101	30252077	30252177	1.19E-46	187	100	100	11	30252127
VuTC20322_1298_1248_1348	CM045819.1	1	101	30252091	30252191	1.19E-46	187	100	100	11	30252141
VuTC20819_606_556_656	CM045819.1	16	101	36813220	36813305	2.59E-38	159	100	85	11	36813255
VuTC21295_283_233_333	CM045819.1	1	61	2833591	2833531	2.05E-24	113	100	100	11	2833541
VuTC21395_469_419_519	CM045819.1	1	101	4638317	4638217	1.19E-46	187	100	100	11	4638267
VuTC21543_375_325_425	CM045819.1	1	101	6875160	6875260	1.19E-46	187	100	100	11	6875210
VuTC21642_866_816_916	CM045819.1	1	101	9131549	9131649	1.19E-46	187	100	100	11	9131599
VuTC21831_6083_6033_6133	CM045819.1	1	101	15796860	15796960	1.19E-46	187	100	100	11	15796910
VuTC22119_491_441_541	CM045809.1	28	101	41626966	41626893	1.21E-31	137	100	100	1	41626943
VuTC22319_438_388_488	CM045818.1	1	101	24046242	24046142	1.19E-46	187	100	100	10	24046192
VuTC22319_77_27_127	CM045818.1	1	101	24046603	24046503	1.19E-46	187	100	100	10	24046553
VuTC23058_1028_978_1078	CM045812.1	15	74	36013342	36013401	7.36E-24	111	100	86	4	36013378
VuTC24042_2561_2511_2583	CM045812.1	1	73	46439531	46439603	2.76E-31	135	100	100	4	46439581
VuTC24699_1593_1543_1643	CM045812.1	1	101	51636425	51636325	5.53E-45	182	99.01	100	4	51636375
VuTC25024_1324_1274_1374	CM045812.1	1	101	53823803	53823703	1.19E-46	187	100	100	4	53823753
VuTC25248_8323_8273_8373	CM045815.1	1	101	7578117	7578217	1.19E-46	187	100	100	7	7578167
VuTC25312_4994_4944_5044	CM045813.1	1	101	33147291	33147391	1.19E-46	187	100	100	5	33147341
VuTC25312_5309_5259_5359	CM045813.1	1	101	33147606	33147706	2.57E-43	176	98.02	100	5	33147656
VuTC25355_209_159_259	CM045813.1	32	96	31562818	31562882	1.22E-26	121	100	95	5	31562837
VuTC25355_209_159_259	CM045813.1	1	36	31562321	31562356	1.62E-10	67.6	100	95	5	31562371
VuTC25491_6046_5996_6096	CM045813.1	1	101	20955499	20955599	1.2E-41	171	97.03	100	5	20955549

VuTC25856_1400_1350_1450	CM045813.1	1	101	13658717	13658617	1.19E-46	187	100	100	5	13658667
VuTC26171_1046_996_1096	CM045813.1	1	101	10365333	10365233	1.19E-46	187	100	100	5	10365283
VuTC27187_1237_1187_1287	CM045813.1	1	101	2923281	2923381	1.19E-46	187	100	100	5	2923331
VuTC27441_1674_1624_1724	CM045813.1	32	101	1533680	1533611	2.03E-29	130	100	100	5	1533661
VuTC28152_1002_952_1052	CM045809.1	1	71	41561653	41561723	5.65E-30	132	100	100	1	41561703
VuTC28154_1391_1341_1441	CM045809.1	1	100	41658663	41658762	4.28E-46	185	100	99	1	41658713
VuTC28154_1530_1480_1580	CM045809.1	1	101	41659267	41659367	1.19E-46	187	100	100	1	41659317
VuTC28154_1646_1596_1696	CM045809.1	1	101	41659383	41659483	1.19E-46	187	100	100	1	41659433
VuTC28154_4577_4527_4627	CM045809.1	1	101	41669342	41669442	1.19E-46	187	100	100	1	41669392
VuTC28154_4595_4545_4645	CM045809.1	1	101	41669360	41669460	1.19E-46	187	100	100	1	41669410
VuTC28154_4614_4564_4664	CM045809.1	1	101	41669379	41669479	1.19E-46	187	100	100	1	41669429
VuTC28154_4619_4569_4669	CM045809.1	1	101	41669384	41669484	1.19E-46	187	100	100	1	41669434
VuTC28154_4769_4719_4819	CM045809.1	1	101	41669533	41669633	1.19E-46	187	100	100	1	41669583
VuTC28155_2560_2510_2610	CM045809.1	1	101	41703835	41703735	1.19E-46	187	100	100	1	41703785
VuTC28163_1003_953_1053	CM045809.1	1	101	41827056	41826956	1.19E-46	187	100	100	1	41827006
VuTC28164_1018_968_1068	CM045809.1	1	97	41849398	41849494	1.99E-44	180	100	96	1	41849448
VuTC28164_974_924_1024	CM045809.1	1	101	41849354	41849454	1.19E-46	187	100	100	1	41849404
VuTC28165_2013_1963_2063	CM045809.1	29	101	41877116	41877044	4.37E-31	135	100	100	1	41877094
VuTC28165_2563_2513_2613	CM045809.1	1	64	41871680	41871617	4.4E-26	119	100	100	1	41871630
VuTC28165_2595_2545_2645	CM045809.1	33	101	41871525	41871457	7.31E-29	128	100	100	1	41871507
VuTC28165_3265_3215_3315	CM045809.1	1	101	41870199	41870099	1.19E-46	187	100	100	1	41870149
VuTC28181_1427_1377_1477	CM045809.1	1	101	41934837	41934937	1.19E-46	187	100	100	1	41934887
VuTC28181_1461_1411_1511	CM045809.1	1	101	41934871	41934971	1.19E-46	187	100	100	1	41934921
VuTC28183_1588_1538_1638	CM045809.1	1	101	42021835	42021935	1.19E-46	187	100	100	1	42021885
VuTC28183_2077_2027_2127	CM045809.1	1	101	42023280	42023380	1.19E-46	187	100	100	1	42023330
VuTC28185_6557_6507_6607	CM045809.1	1	101	42081846	42081746	1.19E-46	187	100	100	1	42081796
VuTC28186_2590_2540_2640	CM045809.1	1	101	42082222	42082122	1.19E-46	187	100	100	1	42082172
VuTC28186_2802_2752_2852	CM045809.1	1	101	42082010	42081910	1.19E-46	187	100	100	1	42081960
VuTC28192_1776_1726_1826	CM045809.1	42	101	42157742	42157683	7.36E-24	111	100	100	1	42157733
VuTC28192_1815_1765_1865	CM045809.1	3	101	42157742	42157644	1.54E-45	183	100	98	1	42157694
VuTC28192_1930_1880_1980	CM045809.1	12	100	42157452	42157364	5.57E-40	165	100	88	1	42157413
VuTC28192_2307_2257_2357	CM045809.1	1	101	42156997	42156897	1.19E-46	187	100	100	1	42156947
VuTC28196_1051_1001_1101	CM045809.1	1	101	42311037	42311137	1.19E-46	187	100	100	1	42311087
VuTC28201_1062_1012_1112	CM045809.1	17	101	42365752	42365668	9.32E-38	158	100	84	1	42365718
VuTC28201_425_375_475	CM045809.1	1	64	42367762	42367699	4.4E-26	119	100	100	1	42367712

VuTC28201_516_466_566	CM045809.1	1	57	42367592	42367536	1.59E-20	100	98.246	100	1	42367542
VuTC28201_680_630_730	CM045809.1	1	101	42367245	42367145	1.19E-46	187	100	100	1	42367195
VuTC28201_76_26_126	CM045809.1	1	78	42369962	42369885	7.26E-34	145	100	77	1	42369912
VuTC28966_1949_1899_1999	CM045809.1	1	101	52172887	52172987	1.19E-46	187	100	100	1	52172937
VuTC29107_1520_1470_1570	CM045809.1	1	101	52792768	52792868	1.19E-46	187	100	100	1	52792818
VuTC29109_1026_976_1076	CM045809.1	1	101	52790172	52790272	1.19E-46	187	100	100	1	52790222
VuTC29109_858_808_908	CM045809.1	1	101	52790004	52790104	1.19E-46	187	100	100	1	52790054
VuTC29349_1348_1298_1398	CM045809.1	1	90	55001235	55001146	7.21E-39	161	98.889	89	1	55001185
VuTC29946_2497_2447_2547	CM045809.1	1	60	59772650	59772709	7.36E-24	111	100	100	1	59772700
VuTC29946_2507_2457_2557	CM045809.1	47	101	59772811	59772865	2.06E-19	97.1	98.182	100	1	59772815
VuTC29946_2525_2475_2575	CM045809.1	29	101	59772811	59772883	2.03E-29	130	98.63	100	1	59772833
VuTC29946_2663_2613_2713	CM045809.1	1	77	59772921	59772997	2.61E-33	143	100	76	1	59772971
VuTC32100_1612_1562_1662	CM045818.1	1	101	29307768	29307868	5.53E-45	182	99.01	100	10	29307818
VuTC32582_1223_1173_1273	CM045818.1	1	101	26767134	26767034	1.19E-46	187	100	100	10	26767084
VuTC34794_4308_4258_4358	CM045812.1	1	101	32392046	32391946	1.19E-46	187	100	100	4	32391996

Table S7. The annotation of transcripts in terms of molecular function.

Transcript _ID[a]	Trait(s)	Description (annotated through OmicsBox)	Enzyme name (EnzCode)	GO Names (GO IDs)	Description (annotated through offline BLASTX)
VuTC15418	Flowering	40S ribosomal protein S19-1	Transferring one-carbon groups(EC:2.1.1)	P:ribosomal small subunit assembly; P:translation; F:RNA binding; F:structural constituent of ribosome; F:methyltransferase activity; C:cytosolic small ribosomal subunit(P:GO:0000028; P:GO:0006412; F:GO:0003723; F:GO:0003735; F:GO:0008168; C:GO:0022627)	40S ribosomal protein S19
VuTC28165	Flowering	5'-3' exoribonuclease 3-like isoform X2	Acting on ester bonds; Acting on ester bonds(EC:3.1.13; EC:3.1.15)	P:nuclear-transcribed mRNA catabolic process; P:mRNA processing; P:RNA phosphodiester bond hydrolysis, exonucleolytic; F:RNA binding; F:5'-3' exoribonuclease activity; F:zinc ion binding; C:nucleus(P:GO:0000956; P:GO:0006397; P:GO:0090503; F:GO:0003723; F:GO:0004534; F:GO:0008270; C:GO:0005634)	5'-3' exoribonuclease 4 /Zinc finger CCHC-type
VuTC28192	Flowering	arabinosyltransferase XEG113	Glycosyltransferases(EC:2.4.2)	P:protein glycosylation; P:cell wall pectin biosynthetic process; F:arabinosyltransferase activity; C:membrane(P:GO:0006486; P:GO:0052325; F:GO:0052636; C:GO:0016020)	arabinosyltransferase XEG113 /Nucleotide-diphospho-sugar transferase/ rhamnogalacturonan II specific xylosyltransferase
VuTC31155	Flowering	bromodomain adjacent to zinc finger domain protein 1A-like	Not Available	Not Available	bromodomain adjacent to zinc finger domain protein 1A-like…,

					golgin subfamily A member 6-like protein 2 [Vigna unguiculata,
VuTC18323	Flowering	Caffeoylshikimate esterase	Acting on ester bonds; 2-acylglycerol O-acyltransferase(EC:3.1; EC:2.3.1.22)	F:2-acylglycerol O-acyltransferase activity; F:lipase activity; C:membrane(F:GO:0003846; F:GO:0016298; C:GO:0016020)	Caffeoylshikimate esterase/Serine aminopeptidase S33 /lysophospholipase
VuTC06771	Flowering	callose synthase 12	1,3-beta-glucan synthase(EC:2.4.1.34)	P:(1->3)-beta-D-glucan biosynthetic process; P:regulation of cell shape; P:cell wall organization; F:1,3-beta-D-glucan synthase activity; C:1,3-beta-D-glucan synthase complex(P:GO:0006075; P:GO:0008360; P:GO:0071555; F:GO:0003843; C:GO:0000148)	Callose synthase, 1-3-beta-glucan synthase subunit FKS1-like domain-1
VuTC06321	Flowering	Cytochrome b5	cytochrome-b5 reductase(EC:1.6.2.2)	F:cytochrome-b5 reductase activity, acting on NAD(P)H; F:heme binding; F:metal ion binding; C:membrane(F:GO:0004128; F:GO:0020037; F:GO:0046872; C:GO:0016020)	cytochrome B5
VuTC28164	Flowering	D-aminoacyl-tRNA deacylase	D-aminoacyl-tRNA deacylase(EC:3.1.1.96)	P:D-amino acid catabolic process; P:aminoacyl-tRNA metabolism involved in translational fidelity; F:metal ion binding; F:D-aminoacyl-tRNA deacylase activity(P:GO:0019478; P:GO:0106074; F:GO:0046872; F:GO:0051499)	D-aminoacyl-tRNA deacylase
VuTC01074	Flowering	heat shock cognate protein 80	nucleoside-triphosphate phosphatase(EC:3.6.1.15)	P:protein folding; P:cellular response to heat; P:protein stabilization; F:ATP binding; F:ATP hydrolysis activity; F:unfolded protein binding; F:ATP-dependent protein folding chaperone;	Heat Shock protein 81,83,90

				C:cytosol; C:plasma membrane; C:protein-containing complex; C:perinuclear region of cytoplasm(P:GO:0006457; P:GO:0034605; P:GO:0050821; F:GO:0005524; F:GO:0016887; F:GO:0051082; F:GO:0140662; C:GO:0005829; C:GO:0005886; C:GO:0032991; C:GO:0048471)	
VuTC31327	Flowering	histone-lysine N-methyltransferase ATX3	Transferring one-carbon groups(EC:2.1.1)	P:regulation of transcription by RNA polymerase II; P:histone methylation; F:histone lysine N-methyltransferase activity; F:metal ion binding; C:Set1C/COMPASS complex(P:GO:0006357; P:GO:0016571; F:GO:0018024; F:GO:0046872; C:GO:0048188)	Histone-lysine N-methyltransferase
VuTC17024	Flowering	hydroxyisourate hydrolase-like isoform X1	Glycosylases(EC:3.2.1)	P:carbohydrate metabolic process; F:hydrolase activity, hydrolyzing O-glycosyl compounds(P:GO:0005975; F:GO:0004553)	beta-glucosidase/hydroxyisourate hydrolase
VuTC26171	Flowering	hypothetical protein VIGAN_05139400, partial	Not Available	C:membrane; C:membrane(C:GO:0016020; C:GO:0016020)	lysine ketoglutarate reductase trans-splicing-like protein…, pentatricopeptide repeat-containing protein [Citrus sinensis]
VuTC08154	Flowering	hypothetical protein VIGAN_UM059100, partial	Not Available	F:nucleic acid binding; F:zinc ion binding(F:GO:0003676; F:GO:0008270)	hypothetical protein LR48_Vigan06g067900, uncharacterized protein HKW66_Vig0200390

VuTC20819	Flowering	kinesin-like protein KIN-13B	Not Available	P:microtubule-based movement; P:microtubule depolymerization; F:microtubule motor activity; F:ATP binding; F:microtubule binding; C:microtubule(P:GO:0007018; P:GO:0007019; F:GO:0003777; F:GO:0005524; F:GO:0008017; C:GO:0005874)	kinesin-like protein/Kinesin motor domain
VuTC28201	Flowering	pectin acetylesterase 8-like	Acting on ester bonds(EC:3.1.1)	P:cell wall organization; F:pectin acetylesterase activity; C:extracellular region(P:GO:0071555; F:GO:0052793; C:GO:0005576)	Pectin acetylesterase 8 /Alpha/Beta hydrolase fold /ribosome maturation protein SDO1
VuTC28196	Flowering	pentatricopeptide repeat-containing protein At1g77360, mitochondrial-like isoform X2	Not Available	Not Available	pentatricopeptide repeat-containing protein
VuTC28155	Flowering	pentatricopeptide repeat-containing protein At3g12770	Acting on ester bonds(EC:3.1)	P:nucleic acid phosphodiester bond hydrolysis; F:endonuclease activity; F:zinc ion binding(P:GO:0090305; F:GO:0004519; F:GO:0008270)	structure-specific endonuclease subunit SLX1/pentatricopeptide repeat-containing protein
VuTC25312	Flowering	pentatricopeptide repeat-containing protein At4g33990	Acting on ester bonds; nucleoside-triphosphate phosphatase(EC:3.1; EC:3.6.1.15)	P:nucleic acid phosphodiester bond hydrolysis; F:endonuclease activity; F:ATP binding; F:zinc ion binding; F:ATP hydrolysis activity(P:GO:0090305; F:GO:0004519; F:GO:0005524; F:GO:0008270; F:GO:0016887)	structure-specific endonuclease subunit SLX1 /Pentatricopeptide repeat-containing protein
VuTC00230	Flowering	Peroxidase 4	peroxidase(EC:1.11.1.7)	P:response to oxidative stress; P:hydrogen peroxide catabolic process; P:cellular oxidant detoxification; F:heme binding;	Peroxidase 4

				F:metal ion binding; F:lactoperoxidase activity; C:extracellular region(P:GO:0006979; P:GO:0042744; P:GO:0098869; F:GO:0020037; F:GO:0046872; F:GO:0140825; C:GO:0005576)	
VuTC28966	Flowering	phosphatidylinositol glycan anchor biosynthesis class U protein	Not Available	P:attachment of GPI anchor to protein; P:protein localization to cell surface; C:GPI-anchor transamidase complex(P:GO:0016255; P:GO:0034394; C:GO:0042765)	phosphatidylinositol glycan anchor biosynthesis class U protein
VuTC04837	Flowering	phosphoenolpyruvate carboxylase	phosphoenolpyruvate carboxylase; phosphoenolpyruvate carboxykinase (GTP)(EC:4.1.1.31; EC:4.1.1.32)	P:tricarboxylic acid cycle; P:carbon fixation; P:photosynthesis; P:leaf development; F:phosphoenolpyruvate carboxylase activity; C:cytosol(P:GO:0006099; P:GO:0015977; P:GO:0015979; P:GO:0048366; F:GO:0008964; C:GO:0005829)	phosphoenolpyruvate carboxylase
VuTC14443	Flowering	Photosystem II PsbX	Oxidoreductases(EC:1)	P:photosynthesis; F:oxidoreductase activity; C:photosystem II(P:GO:0015979; F:GO:0016491; C:GO:0009523)	Photosystem II PsbX
VuTC01110	Flowering	photosystem II stability/assembly factor HCF136, chloroplastic	Not Available	C:chloroplast(C:GO:0009507)	photosystem II stability/assembly factor HCF136
VuTC28152	Flowering	plant UBX domain-containing protein 7	Not Available	P:proteasome-mediated ubiquitin-dependent protein catabolic process; F:protein-macromolecule adaptor activity; F:ubiquitin binding; C:nucleus(P:GO:0043161; F:GO:0030674; F:GO:0043130; C:GO:0005634)	Plant UBX domain-containing protein

VuTC29946	Flowering	plasma membrane ATPase 4	H(+)-exporting diphosphatase; P-type H(+)-exporting transporter; nucleoside-triphosphate phosphatase(EC:7.1.3.1; EC:7.1.2.1; EC:3.6.1.15)	P:regulation of intracellular pH; P:proton export across plasma membrane; F:ATP binding; F:P-type proton-exporting transporter activity; F:ATP hydrolysis activity; C:plasma membrane(P:GO:0051453; P:GO:0120029; F:GO:0005524; F:GO:0008553; F:GO:0016887; C:GO:0005886)	Plasma membrane ATPase
VuTC28185	Flowering	probable apyrase 7	nucleoside diphosphate phosphatase(EC:3.6.1.6)	P:nucleoside diphosphate catabolic process; F:ATP binding; F:nucleoside diphosphate phosphatase activity; C:membrane(P:GO:0009134; F:GO:0005524; F:GO:0017110; C:GO:0016020)	KAG2408970.1 apyrase 7
VuTC28186	Flowering	probable apyrase 7	nucleoside diphosphate phosphatase(EC:3.6.1.6)	P:nucleoside diphosphate catabolic process; F:ATP binding; F:nucleoside diphosphate phosphatase activity; C:membrane(P:GO:0009134; F:GO:0005524; F:GO:0017110; C:GO:0016020)	Apyrase 7
VuTC02166	Flowering	probable E3 ubiquitin-protein ligase RHG1A	Transferases(EC:2)	P:protein ubiquitination; F:metal ion binding; F:ubiquitin protein ligase activity(P:GO:0016567; F:GO:0046872; F:GO:0061630)	E3 ubiquitin-protein ligase RHG1A/ HIP1/X1/X2/X3
VuTC22119	Flowering	Putative isoprenylcysteine alpha-carbonyl methylesterase ICMEL1	arylformamidase; Acting on ester bonds(EC:3.5.1.9; EC:3.1.1)	F:arylformamidase activity; F:prenylcysteine methylesterase activity; C:Golgi membrane; C:endoplasmic reticulum membrane(F:GO:0004061; F:GO:0010296; C:GO:0000139; C:GO:0005789)	isoprenylcysteine alpha-carbonyl methylesterase ICME/ prenylcysteine methylesterase /Carboxylesterase

VuTC28154	Flowering	putative phospholipid-transporting ATPase 9	P-type phospholipid transporter; nucleoside-triphosphate phosphatase(EC:7.6.2.1; EC:3.6.1.15)	P:phospholipid translocation; F:magnesium ion binding; F:ATP binding; F:ATP hydrolysis activity; F:ATPase-coupled intramembrane lipid transporter activity; C:plasma membrane(P:GO:0045332; F:GO:0000287; F:GO:0005524; F:GO:0016887; F:GO:0140326; C:GO:0005886)	phospholipid-transporting ATPase 9 /calcium-transporting ATPase /autoinhibited Ca2+/ATPase II /P-type ATPase
VuTC34794	Flowering	Retrovirus-related Pol polyprotein from transposon TNT 1-94	Not Available	P:DNA integration; F:nucleic acid binding(P:GO:0015074; F:GO:0003676)	Beta-galactosidase , Copia protein, protein kinase domain-containing protein, retrovirus-related pol polyprotein from transposon RE1 [Citrus.
VuTC09869	Flowering	RNA polymerase sigma factor sigA	Not Available	P:cellular response to redox state; P:cellular response to light stimulus; P:photosystem stoichiometry adjustment; P:regulation of DNA-templated transcription initiation; F:DNA binding; F:sigma factor activity; C:chloroplast(P:GO:0071461; P:GO:0071482; P:GO:0080005; P:GO:2000142; F:GO:0003677; F:GO:0016987; C:GO:0009507)	RNA polymerase sigma subunit 1
VuTC09870	Flowering	RNA polymerase sigma factor sigA	Not Available	P:cellular response to redox state; P:cellular response to light stimulus; P:photosystem stoichiometry adjustment; P:regulation of DNA-templated transcription initiation; F:DNA binding; F:sigma factor activity;	RNA polymerase sigma subunit 1 , sigma factor A

				C:chloroplast(P:GO:0071461; P:GO:0071482; P:GO:0080005; P:GO:2000142; F:GO:0003677; F:GO:0016987; C:GO:0009507)	
VuTC09871	Flowering	RNA polymerase sigma factor sigA	Not Available	P:cellular response to redox state; P:cellular response to light stimulus; P:photosystem stoichiometry adjustment; P:regulation of DNA-templated transcription initiation; F:DNA binding; F:sigma factor activity; C:chloroplast(P:GO:0071461; P:GO:0071482; P:GO:0080005; P:GO:2000142; F:GO:0003677; F:GO:0016987; C:GO:0009507)	RNA polymerase sigma factor sigA, RNA polymerase sigma subunit 1
VuTC32100	Flowering	RNA pseudouridine synthase 4, mitochondrial	pseudouridylate synthase; Intramolecular transferases(EC:4.2.1.70; EC:5.4)	P:enzyme-directed rRNA pseudouridine synthesis; F:RNA binding; F:pseudouridylate synthase activity; F:pseudouridine synthase activity(P:GO:0000455; F:GO:0003723; F:GO:0004730; F:GO:0009982)	RNA pseudouridine synthase 4
VuTC29107	Flowering	serine/threonine-protein kinase AFC2 isoform X1	dual-specificity kinase(EC:2.7.12.1)	P:mRNA processing; P:protein phosphorylation; F:protein serine/threonine kinase activity; F:protein serine/threonine/tyrosine kinase activity; F:ATP binding; C:nucleus(P:GO:0006397; P:GO:0006468; F:GO:0004674; F:GO:0004712; F:GO:0005524; C:GO:0005634)	serine/threonine-protein kinase /LAMMER-type protein kinase
VuTC29109	Flowering	serine/threonine-protein kinase AFC2 isoform X3	Transferring phosphorus-containing groups(EC:2.7.1)	P:protein phosphorylation; F:protein kinase activity; F:ATP binding(P:GO:0006468; F:GO:0004672; F:GO:0005524)	Serine/threonine-protein kinase /LAMMER-type protein kinase

VuTC20322	Flowering	silicon efflux transporter LSI3	Not Available	P:transmembrane transport; C:membrane(P:GO:0055085; C:GO:0016020)	silicon efflux transporter LSI3 or LSI2/ arsenical pump membrane protein
VuTC22319	Flowering	STS14 protein	Not Available	Not Available	STS14 protein / pathogenesis-related protein 1
VuTC28183	Flowering	subtilisin-like protease SBT3.5	Acting on peptide bonds (peptidases)(EC:3.4.21)	P:proteolysis; P:response to symbiotic fungus; F:serine-type endopeptidase activity; C:membrane(P:GO:0006508; P:GO:0009610; F:GO:0004252; C:GO:0016020)	Subtilisin-like protease/ Peptidase S8
VuTC15414	Flowering	THO complex subunit 1	Not Available	P:DNA replication; P:mRNA export from nucleus; P:regulation of DNA-templated transcription elongation; C:THO complex part of transcription export complex; C:GINS complex; C:membrane(P:GO:0006260; P:GO:0006406; P:GO:0032784; C:GO:0000445; C:GO:0000811; C:GO:0016020)	THO complex subunit 1
VuTC15693	Flowering	TMV resistance protein N-like	Not Available	P:defense response; P:signal transduction; F:ADP binding(P:GO:0006952; P:GO:0007165; F:GO:0043531)	TMV resistance protein
VuTC28163	Flowering	translin-associated protein X isoform X1	Not Available	F:sequence-specific DNA binding; C:nucleus(F:GO:0043565; C:GO:0005634)	Translin-associated protein X
VuTC04786	Flowering	Two-component response regulator ARR3	Transferring phosphorus-containing groups(EC:2.7)	P:phosphorelay signal transduction system; P:cytokinin-activated signaling pathway; P:phosphorylation; F:kinase activity(P:GO:0000160;	two-component response regulator ARR5/ ARR3/ARR4/ARR6/ARR17, Signal

				P:GO:0009736; P:GO:0016310; F:GO:0016301)	transduction response regulator receiver domain
VuTC02300	Flowering	ubinuclein-1 isoform X3	Not Available	P:chromatin organization; C:nucleus(P:GO:0006325; C:GO:0005634)	Ubinuclein-1 protein
VuTC25024	Flowering	UDP-glycosyltransferase 75D1-like protein	Glycosyltransferases(EC:2.4.1)	F:quercetin 3-O-glucosyltransferase activity; F:quercetin 7-O-glucosyltransferase activity(F:GO:0080043; F:GO:0080044)	Crocetin glucosyltransferase
VuTC28181	Flowering	uncharacterized protein LOC124826145 isoform X2	Not Available	Not Available	Uncharacterized protein HKW66_Vig0038020
VuTC21295	Flowering, Maturity	probable aldo-keto reductase 2	Acting on the CH-OH group of donors(EC:1.1.1)	F:aldo-keto reductase (NADP) activity; C:cytoplasm(F:GO:0004033; C:GO:0005737)	aldo-keto reductase /NADP-dependent oxidoreductase/auxin-induced atb2
VuTC05378	Maturity	4-coumarate--CoA ligase-like 7	Forming carbon-sulfur bonds(EC:6.2)	F:CoA-ligase activity; C:membrane(F:GO:0016405; C:GO:0016020)	coumarate-CoA ligase-like 7, AMP-dependent synthetase/ligase
VuTC08788	Maturity	apoptosis-inducing factor homolog A-like	electron-transferring-flavoprotein dehydrogenase(EC:1.5.5.1)	P:respiratory electron transport chain; F:electron-transferring-flavoprotein dehydrogenase activity; F:flavin adenine dinucleotide binding; C:cytoplasm(P:GO:0022904; F:GO:0004174; F:GO:0050660; C:GO:0005737)	Apoptosis-inducing factor A , External alternative NAD(P)H-ubiquinone oxidoreductase, AMID-like mitochondrial oxidoreductase
VuTC07229	Maturity	chromatin assembly factor 1 subunit FAS1	Not Available	P:nucleosome assembly; C:nucleus; C:membrane; C:CAF-1 complex(P:GO:0006334; C:GO:0005634; C:GO:0016020; C:GO:0033186)	Chromatin assembly factor 1 subunit FAS1

VuTC29349	Maturity	cingulin-like protein 1 isoform X1	Not Available	Not Available	cingulin-like protein 1 /outer dense fiber protein 2 /tropomyosin/shootin-1/spindle pole body component 110
VuTC34182	Maturity	cysteine-rich receptor-like protein kinase 29	Transferring phosphorus-containing groups(EC:2.7.1)	P:protein phosphorylation; F:protein serine/threonine kinase activity; F:ATP binding; C:plasma membrane(P:GO:0006468; F:GO:0004674; F:GO:0005524; C:GO:0005886)	serine/threonine-protein kinase PBS1 /Cysteine-rich receptor-like protein
VuTC21642	Maturity	hypothetical protein LR48_Vigan10s001000	Not Available	C:membrane(C:GO:0016020)	hypothetical protein LR48_Vigan02g061500, uncharacterized protein HKW66_Vig0183570
VuTC32582	Maturity	membrin-11-like	Not Available	P:vesicle fusion; P:protein transport; F:SNARE binding; F:SNAP receptor activity; C:endoplasmic reticulum membrane; C:Golgi apparatus; C:ER to Golgi transport vesicle membrane; C:SNARE complex; C:late endosome membrane(P:GO:0006906; P:GO:0015031; F:GO:0000149; F:GO:0005484; C:GO:0005789; C:GO:0005794; C:GO:0012507; C:GO:0031201; C:GO:0031902)	Bos1/membrin family Qb-SNARE protein, golgi SNAP receptor complex member 2, GOSR2/Membrin/Bos1 ,membrin-11, V-SNARE_C domain-containing protein [Cephalotus follicularis]
VuTC06910	Maturity	No annotation avalable	Not Available	Not Available	Not Available
VuTC27441	Maturity	oligopeptide transporter 4-like	Not Available	P:protein transport; P:oligopeptide transmembrane transport; F:oligopeptide transmembrane transporter activity; C:membrane(P:GO:0015031;	oligopeptide transporter 2/4 /Isp4-like protein

				P:GO:0035672; F:GO:0035673; C:GO:0016020)	
VuTC31321	Maturity	pentatricopeptide repeat-containing protein At4g18975, chloroplastic-like	Not Available	F:zinc ion binding(F:GO:0008270)	B-box-type zinc finger [Vigna unguiculata], Pentatricopeptide repeat-containing protein [Actinidia chinens., PPR containing plant-like protein [Medicago truncatula],
VuTC21395	Maturity	preprotein translocase secA family protein	Transferases(EC:2)	F:transferase activity; F:metal ion binding; F:catalytic activity, acting on a protein; C:membrane(F:GO:0016740; F:GO:0046872; F:GO:0140096; C:GO:0016020)	ubiquitin-protein ligase TRIM32
VuTC18444	Maturity	probable choline kinase 2 isoform X1	ethanolamine kinase; choline kinase(EC:2.7.1.82; EC:2.7.1.32)	P:phosphatidylethanolamine biosynthetic process; P:CDP-choline pathway; P:phosphorylation; F:choline kinase activity; F:ethanolamine kinase activity; C:cytoplasm(P:GO:0006646; P:GO:0006657; P:GO:0016310; F:GO:0004103; F:GO:0004305; C:GO:0005737)	casein kinase (serine/threonine/tyrosine protein kinase) [Pisu…, choline kinase [Glycine max], choline/ethanolamine kinase [Trifolium pratense], Ethanolamine kinase 2, GmCK1p [Glycine max] [Glycine soja], putative phosphotransferase with an alcohol group as acceptor...
VuTC12308	Maturity	probable protein phosphatase 2C 27	protein-serine/threonine phosphatase(EC:3.1.3.16)	P:protein dephosphorylation; F:myosin phosphatase activity; F:metal ion binding(P:GO:0006470;	protein phosphatase 2C 27 ,protein phosphatase 2C 54

				F:GO:0017018; F:GO:0046872)	
VuTC02283	Maturity	probable receptor-like serine/threonine-protein kinase At5g57670	receptor protein-tyrosine kinase(EC:2.7.10.1)	P:peptidyl-tyrosine phosphorylation; F:protein serine/threonine kinase activity; F:transmembrane receptor protein tyrosine kinase activity; F:ATP binding; C:plasma membrane(P:GO:0018108; F:GO:0004674; F:GO:0004714; F:GO:0005524; C:GO:0005886)	Receptor-like cytosolic serine/threonine-protein kinase RBK2/PBS1
VuTC16925	Maturity	protein fluG	Hydrolases; glutamine synthetase(EC:3; EC:6.3.1.2)	P:glutamine biosynthetic process; F:glutamate-ammonia ligase activity; F:hydrolase activity(P:GO:0006542; F:GO:0004356; F:GO:0016787)	protein fluG /Metal-dependent hydrolase /nodulinor glutamate-ammonia ligase/glutamine synthetase
VuTC27187	Maturity	replication factor A1	Not Available	Not Available	DNA-binding protein-related [Musa troglodytarum], Drought-responsive family protein [Hibiscus syriacus], Nucleic acid-binding, OB-fold containing protein [Parasponia…
VuTC21831	Maturity	Retrovirus-related Pol polyprotein from transposon TNT 1-94	Not Available	P:DNA integration; F:nucleic acid binding; F:zinc ion binding; F:hydrolase activity; F:racemase and epimerase activity, acting on amino acids and derivatives(P:GO:0015074; F:GO:0003676; F:GO:0008270; F:GO:0016787; F:GO:0016855)	copia protein (gag-int-pol protein), Cysteine-rich RLK (receptor-like protein kinase) 8, flavonol sulfotransferase-like protein , GAG-pre-integrase domain [Arabidopsis suecica], polyprotein, Integrase catalytic core

					[Arabidopsis suecica],Retrotransposon Copia-like N-terminal [Arabidopsis suecica], Ribonuclease H domain [Arabidopsis suecica], RmlC-like cupins superfamily protein [Prunus dulcis], wall-associated kinase 2, Zinc finger CCHC-type [Arabidopsis thaliana x Arabidopsis…
VuTC33036	Maturity	U-box domain-containing protein 5	Transferases(EC:2)	P:protein ubiquitination; F:ubiquitin-protein transferase activity; C:nucleus; C:cytoplasm(P:GO:0016567; F:GO:0004842; C:GO:0005634; C:GO:0005737)	STIP1 homology and U-box containing protein/U-box domain-containing protein 5
VuTC26129	Maturity	WD repeat-containing protein 48	Not Available	P:double-strand break repair via homologous recombination; P:protein deubiquitination; F:ubiquitin binding(P:GO:0000724; P:GO:0016579; F:GO:0043130)	WD repeat-containing protein 48 /ribosome assembly protein 4
VuTC25248	Maturity, Seed weight	Retrovirus-related Pol polyprotein from transposon TNT 1-94	Acting on ester bonds(EC:3.1)	P:DNA integration; P:RNA phosphodiester bond hydrolysis; F:RNA binding; F:ribonuclease activity(P:GO:0015074; P:GO:0090501; F:GO:0003723; F:GO:0004540)	integrase core domain containing protein, Protein ENHANCED DISEASE RESISTANCE 2, Retrovirus-related Pol polyprotein from transposon RE1 [Glycin..

VuTC25856	Maturity, Seed weight	transcription factor bHLH149	Not Available	P:regulation of DNA-templated transcription; P:response to stress; P:response to oxygen-containing compound; F:transcription cis-regulatory region binding; F:protein dimerization activity; C:nucleus(P:GO:0006355; P:GO:0006950; P:GO:1901700;F:GO:0000976;F:GO:0046983; C:GO:0005634)	transcription factor bHLH147-like, transcription factor PIF3
VuTC19671	Seed weight	40S ribosomal protein S17-4-like	Not Available	P:translation; F:structural constituent of ribosome; C:ribosome; C:ribonucleoprotein complex(P:GO:0006412; F:GO:0003735; C:GO:0005840; C:GO:1990904)	40S ribosomal protein , 40S ribosomal protein S17-2, 40S ribosomal protein S17-3, 40S ribosomal protein S17-4, 40S ribosomal protein S17-4
VuTC25355	Seed weight	60S ribosomal protein L18a	Not Available	P:translation; F:structural constituent of ribosome; C:cytosolic large ribosomal subunit(P:GO:0006412; F:GO:0003735; C:GO:0022625)	60S ribosomal protein L18A /Ribosomal protein 50S-L18Ae/60S-L20/60S-L18A
VuTC16440	Seed weight	alpha-1,6-mannosyl-glycoprotein 2-beta-N-acetylglucosaminyltransferase	alpha-1,6-mannosyl-glycoprotein 2-beta-N-acetylglucosaminyltransferase(EC: 2.4.1.143)	P:protein N-linked glycosylation; P:oligosaccharide biosynthetic process; F:alpha-1,6-mannosylglycoprotein 2-beta-N-acetylglucosaminyltransferase activity; F:metal ion binding; C:Golgi membrane; C:Golgi stack(P:GO:0006487; P:GO:0009312; F:GO:0008455; F:GO:0046872; C:GO:0000139; C:GO:0005795)	N-acetylglucosaminyltransferase
VuTC23058	Seed weight	Apyrase 2	nucleoside diphosphate	P:nucleoside diphosphate catabolic	apyrase / Nucleoside

			phosphatase(EC:3.6.1.6)	process; F:ATP binding; F:nucleoside diphosphate phosphatase activity; C:membrane(P:GO:0009134; F:GO:0005524; F:GO:0017110; C:GO:0016020)	phosphatase GDA1/CD39
VuTC24042	Seed weight	cactin isoform X1	Not Available	P:embryo development ending in seed dormancy; P:mRNA cis splicing, via spliceosome; C:spliceosomal complex; C:cytoplasm(P:GO:0009793; P:GO:0045292; C:GO:0005681; C:GO:0005737)	Cactin protein
VuTC12393	Seed weight	DEAD-box ATP-dependent RNA helicase 27	RNA helicase(EC:3.6.4.13)	P:maturation of LSU-rRNA from tricistronic rRNA transcript (SSU-rRNA, 5.8S rRNA, LSU-rRNA); F:RNA binding; F:RNA helicase activity; F:ATP binding; F:hydrolase activity; C:nucleolus(P:GO:0000463; F:GO:0003723; F:GO:0003724; F:GO:0005524; F:GO:0016787; C:GO:0005730)	DEAD-box ATP-dependent RNA helicase
VuTC12822	Seed weight	DNA topoisomerase 2	Isomerases(EC:5)	P:resolution of meiotic recombination intermediates; P:sister chromatid segregation; F:nucleotide binding; F:DNA topoisomerase activity; F:ion binding; C:nucleus(P:GO:0000712; P:GO:0000819; F:GO:0000166; F:GO:0003916; F:GO:0043167; C:GO:0005634)	DNA topoisomerase 2
VuTC06850	Seed weight	kinesin-like protein KIN-4A isoform X2	Not Available	P:microtubule-based movement; F:microtubule motor activity; F:ATP binding; F:microtubule	Chromosome-associated kinesin /KIF4/ FRA1,

				binding; C:microtubule(P:GO:0007018; F:GO:0003777; F:GO:0005524; F:GO:0008017; C:GO:0005874)	kinesin-like protein KIN-4A kinesin family member 4/7/21/27
VuTC06074	Seed weight	myosin-1	Glycosyltransferases; myosin ATPase(EC:2.4.1; EC:5.6.1.8)	P:protein glycosylation; P:actin filament organization; P:vesicle transport along actin filament; P:flavin-containing compound biosynthetic process; P:UDP-glucosylation; F:microfilament motor activity; F:nucleotide binding; F:UDP-glucose:glycoprotein glucosyltransferase activity; F:metal ion binding; F:actin filament binding; C:actin cytoskeleton(P:GO:0006486; P:GO:0007015; P:GO:0030050; P:GO:0042727; P:GO:0097359; F:GO:0000146; F:GO:0000166; F:GO:0003980; F:GO:0046872; F:GO:0051015; C:GO:0015629)	myosin-1, UDP-glucose:glycoprotein glucosyltransferase
VuTC09951	Seed weight	pentatricopeptide repeat-containing protein At4g25270, chloroplastic	Transferring phosphorus-containing groups; nucleoside-phosphate kinase; adenylate kinase; Acting on peptide bonds (peptidases)(EC:2.7.1; EC:2.7.4.4; EC:2.7.4.3; EC:3.4)	P:protein phosphorylation; P:proteolysis; P:nucleoside monophosphate phosphorylation; P:cell division; F:adenylate kinase activity; F:protein serine/threonine kinase activity; F:ATP binding; F:peptidase activity(P:GO:0006468; P:GO:0006508; P:GO:0046940; P:GO:0051301; F:GO:0004017; F:GO:0004674; F:GO:0005524; F:GO:0008233)	Adenylate kinase, Pentatricopeptide repeat-containing protein, PPR domain-containing protein/PPR_2 domain-containing protein.., putative serine/threonine protein kinase IRE , putative tetratricopeptide-like helical domain-

					containing..
VuTC00580	Seed weight	probable tyrosine-protein phosphatase DSP4 isoform X1	Acting on ester bonds(EC:3.1.3)	P:protein dephosphorylation; F:protein tyrosine/serine/threonine phosphatase activity; C:cytoplasm(P:GO:0006470; F:GO:0008138; C:GO:0005737)	tyrosine-protein phosphatase, Tyrosine-protein phosphatase DSP1
VuTC13707	Seed weight	protein SHOOT GRAVITROPISM 6 isoform X1	Not Available	Not Available	Armadillo-type fold , Coatomer beta subunit, HEAT repeat-containing protein 7A like, Protein SHOOT GRAVITROPISM 6 [Glycine max]
VuTC30884	Seed weight	putative disease resistance protein At3g14460	Not Available	P:defense response; P:response to other organism; F:ADP binding(P:GO:0006952; P:GO:0051707; F:GO:0043531)	disease resistance RPP13-
VuTC24699	Seed weight	rop guanine nucleotide exchange factor 5	Not Available	P:regulation of catalytic activity; F:guanyl-nucleotide exchange factor activity(P:GO:0050790; F:GO:0005085)	rop guanine nucleotide exchange factor 5/ RHO guanyl-nucleotide exchange factor 7
VuTC02299	Seed weight	rust resistance kinase Lr10-like	Transferring phosphorus-containing groups(EC:2.7.1)	P:protein phosphorylation; F:protein serine/threonine kinase activity; F:ATP binding; F:polysaccharide binding; C:membrane(P:GO:0006468; F:GO:0004674; F:GO:0005524; F:GO:0030247; C:GO:0016020)	stress-induced receptor-like kinase, Rust resistance kinase Lr10 , brassinosteroid insensitive 1-associated receptor kinase 1, somatic embryogenesis receptor kinase 4
VuTC21543	Seed weight	TMV resistance protein N-like	Not Available	P:defense response; P:signal transduction; F:ADP binding; C:membrane(P:GO:0006952; P:GO:0007165; F:GO:0043531;	TMV resistance protein

				C:GO:0016020)	
VuTC25491	Seed weight	Ty3/gypsy retrotransposon protein	Acting on peptide bonds (peptidases); RNA-directed DNA polymerase(EC:3.4.23; EC:2.7.7.49)	P:RNA-templated DNA biosynthetic process; P:proteolysis; P:DNA integration; F:nucleic acid binding; F:RNA-directed DNA polymerase activity; F:aspartic-type endopeptidase activity(P:GO:0006278; P:GO:0006508; P:GO:0015074; F:GO:0003676; F:GO:0003964; F:GO:0004190)	uncharacterized protein HKW66_Vig0095200
VuTC01132	Seed weight	WRKY transcription factor 1	Not Available	P:salicylic acid mediated signaling pathway; P:positive regulation of DNA-templated transcription; F:transcription cis-regulatory region binding; F:DNA-binding transcription factor activity; F:zinc ion binding; C:nucleus(P:GO:0009863; P:GO:0045893; F:GO:0000976; F:GO:0003700; F:GO:0008270; C:GO:0005634)	WRKY transcription factor 1/33

[a] The transcript IDs in bold italics are associated with more than one trait.

Table S8. Sub-population wise phenotypic data with mean values for each dataset

		Almora 2020			Almora 2021			Delhi 2020			Delhi 2021		
Accession	Sub-Population	Days to 50% Flowering	Days To 80% Maturity	100-Seed weight	Days to 50% Flowering	Days To 80% Maturity	100-Seed weight	Days to 50% Flowering	Days To 80% Maturity	100-Seed weight	Days to 50% Flowering	Days To 80% Maturity	100-Seed weight
EC18136	1	78	119	7.29	82	121	8.15	62	112	4.5	66	110	7.09
EC18222	1	80	115	8.64	81	120	8.37	62	119	3.37	69	115	5.84
EC1843	1	67	113	7.05	75	120	7.4	52	108	4.55	67	92	5.28
EC615199	1	78	130	7.18	82	130	8.54	82	115	8.06	86	117	5.7
EC615201	1	77	122	7.29	79	115	7.85	62	115	5.52	65	108	7.12
IC129062	1	70	110	7.82	75	115	7.18	64	113	4.45	68	120	5.72
IC129069	1	75	115	9.65	78	121	7.58	63	113	4.67	67	107	6.01
IC129078	1	70	148	8.28	77	131	8.15	66	113	4.75	72	128	5.9
IC129080	1	73	119	9.15	78	125	9.65	66	115	7.17	70	120	7.32
IC129089	1	70	107	8.95	75	113	9.21	78	118	4.6	84	119	6.66
IC129097	1	70	128	9.35	76	115	8.65	53	111	5.14	88	142	6.69
IC129115	1	70	138	7.95	72	110	7.15	59	113	5.35	70	118	6.41
IC141074	1	80	137	8.25	81	123	8.18	54	113	8.98	67	114	5.79
IC15642	1	98	133	7.89	69	105	7.28	82	120	7.34	88	136	5.91
IC15663	1	70	128	9.15	72	116	7.95	62	121	5.03	68	129	4.61
IC15664	1	76	130	9.85	78	114	7.91	62	123	6.2	80	120	7.55
IC16706	1	80	110	8.02	82	112	7.98	64	120	5.63	70	126	6.49
IC16796	1	73	108	7.19	78	115	7.08	64	113	4.63	68	111	6.41
IC16799	1	74	130	8.19	75	126	8.05	72	124	3.83	69	120	5.56
IC273820	1	100	135	8.65	89	132	7.18	86	122	4.58	98	123	0.73
IC298121	1	96	128	7.82	92	128	7.98	87	122	9.2	95	120	5.94
IC311934	1	71	133	7.18	73	116	7.88	93	123	3.91	93	145	6.55
IC342229	1	100	130	7.18	90	129	9.65	63	120	4.7	91	121	5.43
IC342242	1	87	132	8.25	84	130	8.54	60	123	7.45	73	122	7.85

IC343841	1	98	122	8.02	90	137	7.18	86	123	4.58	95	118	5.84
IC350127	1	70	122	7.95	73	112	7.98	86	122	6.27	92	118	5.27
IC350374	1	100	130	7.05	89	129	8.54	82	120	5.84	98	121	6.32
IC350791	1	99	122	7.05	92	139	7.25	82	120	4.48	90	146	4.22
IC351534	1	101	134	8.26	90	136	7.08	82	115	7.7	74	116	6.4
IC352853	1	94	122	8.95	89	125	7.58	52	122	11.31	89	129	5.98
IC352931	1	89	122	7.95	85	120	7.95	81	122	4.79	91	118	3.4
IC352944	1	76	121	9.03	79	125	7.08	82	98	5.55	71	118	6.09
IC353877	1	75	133	7.98	78	128	8.02	82	120	5.41	92	122	2.91
IC369607	1	75	136	7.05	76	112	7.98	82	115	6.01	93	156	6.75
IC373406	1	80	130	8.19	77	128	8.54	86	122	5.3	96	153	5.88
IC423374	1	79	129	7.98	84	128	7.98	64	123	11	89	147	6.28
IC426778	1	76	130	7.18	80	124	7.82	86	122	6.14	78	138	7.17
IC426783	1	96	130	9.16	90	131	7.98	85	125	4.94	87	128	6.14
IC426789	1	74	130	9.16	78	128	8.54	52	123	6.09	92	148	6.81
IC433853	1	81	128	8.61	81	126	7.82	67	122	4.63	72	125	3.83
IC435833	1	74	130	8.02	82	132	7.65	69	114	3.88	87	148	6.2
IC449234	1	72	119	7.65	77	122	7.76	62	113	2.68	71	127	7.2
IC521177	1	81	128	9.14	85	128	8.69	66	87	5.45	71	123	4.59
IC552970	1	74	130	7.19	81	128	8.65	123	137	4.73	112	121	5.87
IC552977	1	71	109	8.96	72	110	7.98	70	115	7.2	72	126	6.86
IC552995	1	90	133	7.05	84	130	8.65	123	137	3.95	144	122	5.72
IC554711	1	69	116	9.56	76	121	8.94	86	126	5.73	88	116	5.77
IC557283	1	71	128	8.06	83	127	8.27	66	128	8.14	71	128	5.71
IC557317	1	101	130	7.05	92	138	7.18	62	123	9.52	69	118	10.79
IC567233	1	81	124	7.64	82	122	7.08	117	134	4.21	98	119	5.77
IC569073	1	72	124	8.47	76	114	9.58	62	122	4.09	67	110	4.66
IC569120	1	92	130	8.49	85	128	7.95	119	135	4.25	89	119	5.26
IC621844	1	91	124	8.65	87	130	8.27	59	95	7.02	99	119	6.04

**Sub-population 1 Mean**		**80.85**	**125.74**	**8.15**	**80.87**	**123.40**	**8.03**	**74.32**	**118.66**	**5.75**	**82.43**	**124.15**	**5.93**
EC114075	2	73	133	7.05	78	126	7.4	86	122	4.72	67	112	5.93
EC18183	2	71	104	7.25	79	112	6.45	47	108	6.11	63	109	5.91
EC615195	2	68	119	8.75	70	109	7.54	62	112	5.67	65	108	4.32
EC934343	2	71	104	8.21	81	127	7.26	66	104	2.19	71	104	5.51
IC112381	2	71	133	9.27	77	126	8.76	62	95	1.9	67	103	4
IC112383	2	76	129	9.65	78	124	6.45	57	95	6.27	65	116	6.43
IC116113	2	74	114	7.58	76	120	8.58	57	90	4.13	64	112	5.74
IC129038	2	70	126	7.19	76	120	7.95	62	105	7.64	65	118	5.46
IC137205	2	76	119	7.25	81	123	8.69	59	111	5.33	67	94	5.69
IC140810	2	81	96	8.62	82	120	8.69	58	95	4.58	70	125	6.57
IC144695	2	76	133	7.95	79	126	7.95	58	92	6.2	65	122	6.06
IC2074	2	66	130	7.61	68	108	8.69	62	115	6.19	65	108	4.41
IC2567	2	78	120	8.54	82	120	8.34	61	112	4.06	70	110	5.77
IC26962	2	75	109	7.26	77	112	7.95	62	123	4.84	63	106	6.2
IC316123	2	76	114	8.25	78	114	8.27	71	123	6.65	65	109	3.9
IC326994	2	81	112	8.25	81	120	8.13	62	105	6.16	67	114	5.99
IC343929	2	73	122	8.02	78	123	7.99	62	112	7.88	92	118	5.09
IC362094	2	82	133	5.65	82	136	7.98	62	123	2.41	65	102	2.98
IC369230	2	78	123	7.31	80	125	7.82	82	120	5.27	70	125	5.47
IC384034	2	76	108	8.19	77	112	8.25	83	121	6.74	64	104	6.05
IC469185	2	72	124	7.25	72	123	7.58	57	115	4.27	63	100	6.07
IC521039	2	84	130	7.45	82	125	7.64	61	61	4.57	66	121	6.15
IC521146	2	83	132	8.41	81	130	7.82	62	107	5.18	66	110	6.44
IC521156	2	74	119	7.79	76	115	7.18	62	108	3.75	66	118	4.73
IC521224	2	84	144	8.14	86	135	7.98	76	113	7.79	72	112	6.75
IC521356	2	70	113	8.16	75	115	9.65	63	84	4.63	68	116	6.37
IC545607	2	71	112	7.96	72	113	8.65	123	137	4.82	118	114	5.89

IC551632	2	82	128	7.05	78	123	7.25	66	113	4.18	70	128	5.56
IC551711	2	81	128	8.17	81	129	8.18	67	133	4.67	72	117	7.48
IC552979	2	76	111	8.24	79	119	8.13	122	136	4.23	113	114	5.76
IC564865	2	100	131	7.05	90	136	7.98	122	136	4.99	98	121	3.96
IC621805	2	81	129	7.19	81	125	7.48	60	101	4.15	67	121	5.86
**Sub-population 2 Mean**		**76.56**	**121.31**	**7.83**	**78.53**	**121.59**	**7.96**	**69.44**	**110.22**	**5.07**	**71.53**	**112.84**	**5.58**
EC16167	3	89	127	7.02	84	127	7.18	48	107	3.48	65	110	7
EC18261	3	101	140	7.05	90	139	8.25	48	107	5.74	53	73	4.15
EC615198	3	80	121	8.12	78	126	9.65	62	52	6.71	55	90	7.25
EC934368	3	52	70	8.55	81	124	8.32	49	70	1.86	52	70	3.77
EC934417	3	45	67	8.62	66	106	8.36	44	67	1.76	45	67	3.26
IC116118	3	70	112	8.96	70	114	7.95	57	60	4.04	64	106	5.21
IC137169	3	73	119	8.65	79	119	8.69	59	64	2.89	55	106	4.79
IC144685	3	80	116	7.98	78	120	8.29	60	101	5.04	63	114	5.63
IC144701	3	78	109	8.68	77	113	8.27	58	111	5.8	67	118	5.45
IC19351	3	73	122	7.15	76	116	8.59	57	114	4.88	72	122	7.19
IC351508	3	78	118	8.54	80	121	8.31	52	113	2.19	55	80	5.5
IC469177	3	71	124	7.28	87	122	8.18	62	91	4.7	55	74	5.24
IC538983	3	71	114	8.74	71	112	9.64	61	113	5.2	65	100	6.12
IC564858	3	101	131	8.49	79	118	7.25	117	134	4.72	113	121	5.87
IC618587	3	78	131	7.98	80	130	7.18	61	92	5.17	63	102	6.04
**Sub-population 3 Mean**		**76**	**114.73**	**8.12**	**78.40**	**120.46**	**8.27**	**59.66**	**93.07**	**4.28**	**62.80**	**96.87**	**5.50**
